# A gap analysis of UK biobank publications reveals SNPs associated with intrinsic subtypes of breast cancer

**DOI:** 10.1016/j.csbj.2024.05.001

**Published:** 2024-05-10

**Authors:** Lisa van den Driest, Patricia Kelly, Alan Marshall, Caroline H. Johnson, Jessica Lasky-Su, Alison Lannigan, Zahra Rattray, Nicholas J.W. Rattray

**Affiliations:** a Strathclyde Institute of Pharmacy and Biomedical Sciences, University of Strathclyde, 161 Cathedral Street, Glasgow G4 0RE, UK; b School of Social and Political Science, University of Edinburgh, Chrystal Macmillan Building, George Square, Edinburgh EH8 9LD, UK; c Yale School of Public Health, Yale University, 60 College Street, New Haven, CT 06510, USA; d Brigham and Women's Hospital and Harvard Medical School, 181 Longwood Ave, Boston, MA 02115, USA; e NHS Lanarkshire, Lanarkshire, Scotland, UK; f Wishaw General Hospital, NHS Lanarkshire, 50 Netherton St, Wishaw ML2 0DP, UK

**Keywords:** Breast cancer, Genomics, SNPs, UK Biobank

## Abstract

Breast cancer is a multifaceted disease and a leading cause of cancer morbidity and mortality in females across the globe. In 2020 alone, 2.3 million women were diagnosed and 685,000 died of breast cancer worldwide. With the number of diagnoses projected to increase to 3 million per year by 2040 it is essential that new methods of detection and disease stratification are sought to decrease this global cancer burden.

Although significant improvements have been made in breast cancer diagnosis and treatment, the prognosis of breast cancer remains poor in some patient groups (i.e. triple negative breast cancer), necessitating research into better patient stratification, diagnosis and drug discovery. The UK Biobank, a comprehensive biomedical and epidemiological database with a wide variety of multiomics data (genomics, proteomics, metabolomics) offers huge potential to uncover groundbreaking discoveries in breast cancer research leading to improved patient stratification. Combining genomic, proteomic, and metabolic profiles of breast cancer in combination with histological classification, can aid treatment decisions through accurate diagnosis and prognosis prediction of tumor behaviour.

Here, we systematically reviewed PubMed publications reporting the analysis of UK Biobank data in breast cancer research. Our analysis of UK Biobank studies in the past five years identified 125 publications, of which 76 focussed on genomic data analysis. Interestingly, only two studies reported the analysis of metabolomics and proteomics data, with none performing multiomics analysis of breast cancer. A meta-analysis of the 76 publications identified 2870 genetic variants associated with breast cancer across 445 genes. Subtype analysis revealed differential genetic alteration in 13 of the 445 genes and the identification of 59 well-established breast cancer genes. in differential pathways. Pathway interaction analyses illuminated their involvement in general cancer biomolecular pathways (e.g. DNA damage repair, Gene expression). While our meta-analysis only measured genetic differences in breast cancer due to current usage of UK Biobank data, minimal multi-omics analyses have been performed and the potential for harnessing multi-omics strategies within the UK Biobank cohort holds promise for unravelling the biological signatures of distinct breast cancer subtypes further in the future.

## Graphical Abstract

**Figure:**
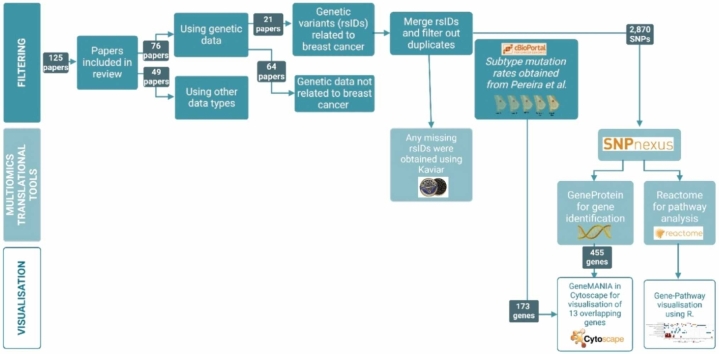

## 1 Introduction

Breast cancer is a global health challenge with an estimate of 2.26 million new cases and 685,000 new deaths recorded worldwide in 2020 [1] and 55,400 new cases and 11,585 new deaths in the UK [2] in 2023 [3]. These stark statistics have led the disease to surpass lung cancer as the leading global cause of cancer incidence in women [1]. The disease is characterized by intricate clinical manifestations and diverse molecular mechanisms relating to disease stage and phenotype. Great strides have been made in developing stratified approaches that are being used to direct therapy. The five PAM50 molecular intrinsic subtypes, luminal A, luminal B, HER2-enriched, basal-like and normal-like have distinct morphological features, biological properties, epidemiological risk factors, prognoses, and response to therapy [4], [5], [6]. The basal-like subtype is for 80% defined as triple-negative breast cancer (TNBC) [7], characterised by the absence of oestrogen receptor (ER), progesterone receptor (PR), and human epidermal growth factor receptor 2 (HER2) expression. Current evidence considers TNBC as a hypernym covering a variety of entities including genetic, transcriptional, histological, and clinical differences [8]. TNBC is associated with poor prognosis and limited treatment options, highlighting the potential to shift from a targeted approach into personalised network-based approaches mapping the differential molecular sub phenotypes of TNBC. Technological breakthroughs have been critical in developing this new shift of understanding in breast cancer stratification and the ability to analyse large sample cohorts, collected in highly similar ways through the development of disease specific biobanks, is one such vital component.

A biobank is a functional unit responsible for the collection, storage and provision of biological samples and their accompanying data, as vital resources for the progression and democratisation of clinical and biomedical research [9]. The collection and storage of biospecimens has been a long-existing practice for over 150 years [10]. More recently, the past 40 years has seen the rapid expansion of biobanking from small, predominantly university-based repositories to institutional or government-sponsored repositories, population-based biobanks and virtual biobanks [11]. This transformation has been driven by the evolving needs of larger research projects requiring higher powered analyses to help stratify disease via rapidly emerging fields such as genomics, proteomics, metabolomics, big data and precision medicine [11]. In the early 2000s, the recognised value of data-driven, population-based studies gave rise to the establishment of the Oxford University based UK Biobank [12]. Since then, the UK Biobank has evolved into a unique research resource containing extensive genomic, phenotypic, health-related and socioeconomic data from ∼500,000 volunteers in the United Kingdom (Table 1) [13]. The pivotal advantage of large-scale population biobanks such as the UK Biobank, is their potential to accelerate discoveries in personalised medicine for the prevention, stratification, diagnosis and monitoring of disease progression and response to treatment, ultimately supporting better disease outcomes [14]. A well-recognized challenge in biomedical sciences is the issue of low statistical power, which can be caused by low sample size in research studies [15]. In such cases, there is a higher likelihood that the true effect size between variables is either zero or very small, rendering it challenging to detect significant findings from population level datasets [15]. The large sample size of the UK Biobank dataset allows for the more precise estimation of effect sizes, increased statistical power, and improves the scalability of research findings. Beyond existing resources (Table 1), additional UK Biobank data will be released in Q4 2023, which will include imaging data, information on well-being, and an expanded release of proteomics data.

**Table 1:** **Overview of the resources contained in the UK Biobank.** The item count stands for the number of data ‘points’ corresponding to the central database. For example, the study involved 100 participants whose height was measured on three separate occasions using identical procedures. As a result, the cumulative item count for the height field in total is 300 observations. A data field corresponds to a specific subcategory or type of data. Item counts are up to date for 21 July 2023.

**Resources**		**Total data items/fields**	
**Summary Data**			
Participants	502,371		
Gender %	273,302 Female (54.4%)	535,991 data items covering 501,495 participants	
	229,069 Male (45.6%)		
Ethnicity %	White (94.4%)	506,136 data items	
	Mixed (0.6%)	3109 data items	
	Asian and Asian British (1.9%)	10,177 data items	
	Black or Black British (1.5%)	8237 data items	
	Chinese (0.3%)	1659 data items	
	Other ethnicities (0.9%)	4703 data items	
	Do not know (0.04%)	223 data items	
	Prefer not to answer (0.3%)	1747 data items	
**Clinical and Health Data**			
Biological sampling	Blood	587,222 data items covering 501,215 participants	
	Urine	3560 items covering 3560 participants	
	Saliva	782 items covering 782 participants	
Health data	Cognitive function	453 data-fields	
	Health outcomes	555 data-fields	
	Physical measure summary	66 data-fields	
	Family history	20 data-fields	
	Lifestyle	461 data-fields	
	Early life and reproductive factors	20 data-fields	
	Image measures	1055 data-fields	
	Socio-demographics	111 data-fields	
	Geographical measures	38 data-fields	
**Multiomic data**			
Genomics	Genotype data	825,927 markers covering 438,427 participants	
	Whole exome sequencing	470,000 participants	
	Whole genome sequencing	200,000 participants	
Proteomics	OLink Protein biomarkers	2923 markers covering 52 749 participants	
Metabolomics	Nightingale NMR biomarkers	249 markers covering 120,000 participants	
**Access and output**			
Costs for accessing datasets	Core data (£3000) (e.g., questionnaires, linked health data)		
	Assay data and enhanced measures (£6000) (e.g., biochemical, and haematological assays, measures, and imputed genotypes)		
	Very large datasets (£9000) (e.g., imaging data, whole genome/exome data)		
Publications per year	2008 (1), 2012 (1), 2013 (6), 2014 (16), 2015 (30), 2017 (173), 2018 (310), 2019 (429), 2020 (664), 2021 (931), 2022 (584)		

**Fig. 1:**
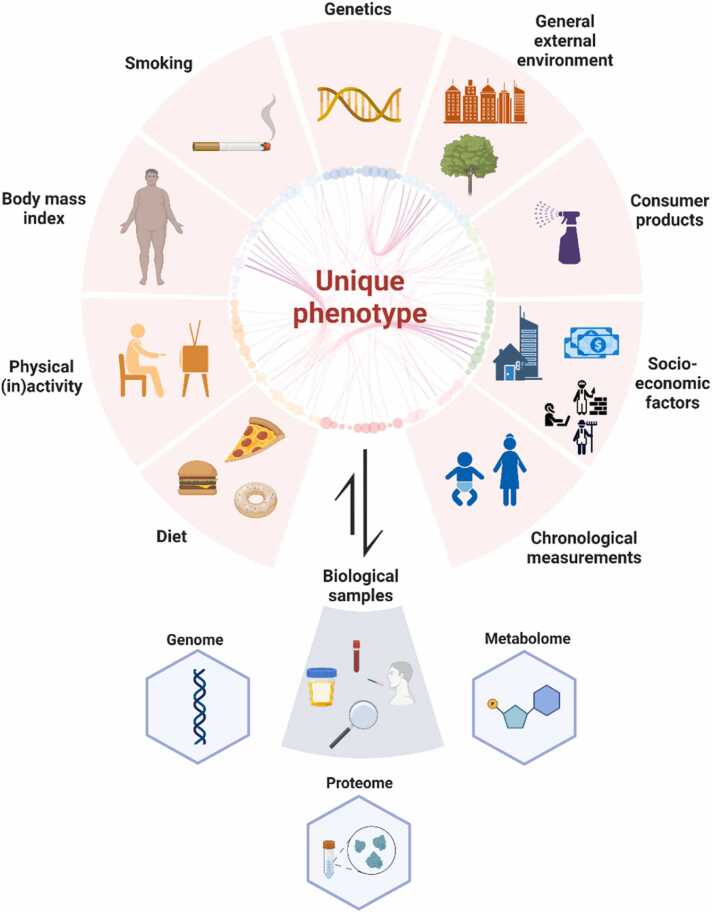
**Personalised Medicine potential of the UK Biobank.** An individual's unique biomolecular phenotype arises from the interplay between nature (hereditary factors) and nurture (environmental factors). This biomolecular phenotype can inform healthcare professionals on an individual’s health and disease status. Created with BioRender.com.

The integration of these additional population level data available through the UK Biobank offers a unique opportunity for large-scale research. This has the potential to transform the current paradigm of conventional medicine based on a generalised ‘one size fits all’ approach to a personalised medicine approach tailored to individual patient profiles. This shift is facilitated by the ability to leverage network-based analysis methods that move beyond a simplistic targeted approach treating different disease subtypes to enable more comprehensive understanding and treatment of complex diseases. The incorporation of genetic, lifestyle, occupation, housing, and environmental data can aid further disease stratification into subpopulations translated by the individual unique phenotype as shown in Fig. 1. By enabling large-scale, data-driven research and personalized medicine, the integration of UK Biobank population data holds significant potential to drive innovation and shape the future of medicine.

To further advance breast cancer research, several additional population-based biobanks have emerged in the recent decades, as indicated in Table 2. The emergence of these biobanks is driven by the growing recognition of their invaluable role as resources for studying genetic, environmental (e.g., pollution levels), and lifestyle factors that contribute to breast cancer development, progression, and response to treatment. By harnessing the power of large-scale population-based studies, scientists can gain deeper insights into the complex mechanisms underlying breast cancer and ultimately improve prevention, diagnosis, and treatment strategies. Upon comparison of the UK Biobank (Table 1) with other population-based biobanks dedicated to breast cancer research (Table 2), we observe that the UK Biobank provides a more comprehensive resource encompassing a wide range of data types, including those related to lifestyle, environmental factors, and particularly multi-omics applications.

**Table 2:** UK Biobank in comparison with other population-based biobanks available for breast cancer research with participant numbers over 100,000.

Biobanks	Launch date	Funding body	Number of participants	Data types	Omics data	Accessibility
*UK Biobank*	2012	Core funders:Wellcome Trust medical charity, Medical Research Council, Department of Health, Scottish Government and the Northwest Regional Development Agency.	500,000 (UK)	Genetic data, lifestyle data, linkage to electronic health-related records, environmental data, biological samples, imaging data, follow-up data.	Genomic, proteomic and metabolomic data.	Researchers can apply to access data through the biobank website.
*Women's Health Initiative*[16]	1992	The National Heart, Lung, and Blood Institute (NHLBI) and the US Department of Health and Human Services.	161,000 postmenopausal women (USA)	Biological samples, including blood, urine, and saliva, and physical exam findings. Breast cancer incidence, risk factors, outcomes, other diseases, and health conditions.	Genomic[17], proteomic[18] and metabolomic data[19].	Researchers can apply to access data and samples through the biobank website.
*Breast Cancer Now Tissue Bank*[20]	1992	Breast Cancer Now, a UK charity dedicated to funding breast cancer research and support services	120,000 samples from 10,000 patients with breast cancer (UK).	Range of tissue samples: tumor tissue, normal breast tissue and blood samples. Clinical data such as tumor stage and grade, treatment history and patient demographics.	Genomic data.	Researchers can apply for access to the tissue bank's resources through an online application process.
*Million Women Study*[21]	1996	Medical Research Council (MRC), Cancer Research UK (CRUK), the UK National Health Service (NHS), and the British Heart Foundation (BHF)	> 1 million women participants (UK).	Breast cancer incidence, risk factors, outcomes, other diseases, and health conditions. Self-reported lifestyle factors such as diet, exercise, smoking, hormone therapy use, medical history.	Genomic data[22].	Researchers can apply to access data and samples through the biobank’s website.
*Estonian Biobank*[23]	2000	Estonian Genome Centre (University of Tartu and the Ministry of Education and Research of Estonia).	> 200,000 participants (Estonia)	Genomic data (genotyping, exome sequencing, whole-genome sequencing), health data (hospital discharge data, prescription data, death registry data, biobank data), and lifestyle data (questionnaire data).	Genomic and some other -omic (proteomic, metabolomic data) However, the availability of -omic data varies depending on the specific study and sample collection within each biobank.	Estonian Biobank provides access to their data and samples for scientific research via a data access application process.
*Breast Cancer Association Consortium (BCAC)*[24]	2005	Various organisations, including Cancer Research UK, the US National Cancer institute, and the European Union.	300,000 participants (September 2021) (Global)	Clinical data and lifestyle factor – depending on the individual study.	Genomic data[25].	To access BCAC data, researchers need to submit a data access request through the consortium website.
*Partners Healthcare Biobank*[26]	2007	Partners Healthcare, a US-based health system and research institution	100,000 participants (USA)	Biospecimens: blood, urine, tissue samples. Clinical data (medical history, and health behaviours).	Genomic data[27].	Researchers can apply to access to the biobank's resources through an online application process.
*FinnGen*[28]	2017	The Finnish government, universities, hospitals, and foundations.	Currently, 330,000 Finnish individuals, the aim is to reach 500,000.	Genomic data (whole-genome sequencing, exome sequencing, genotyping), health data (hospital discharge data, prescription data, cancer registry data, death registry data, biobank data), and lifestyle data (questionnaire data).	Genomic and other -omic (proteomic, metabolomic data) However, the availability of -omic data varies depending on the specific study and sample collection within each biobank.	FinnGen provides access to their data and samples for scientific research via a secure online platform.

## 2 Study aim

In this study, we aim to identify the gaps in breast cancer research utilising the UK Biobank as a resource. We analyse the prevalent data types within the UK Biobank used in breast cancer research, visualise their interconnections, and subsequently determine which are most used in the UK Biobank, visualise how these were linked together and subsequently determine the most frequently utilised data type to assess its contribution to advancing breast cancer research.

## 3 Methods

### 3.1 Data search strategy and selection criteria

A systematic search of the PubMed database (https://pubmed.ncbi.nlm.nih.gov/) was performed in accordance with the Preferred Reporting Items for Systematic Reviews and Meta-Analysis (PRISMA) guidelines [29]. The following search terms were used: “UK Biobank” and “Breast cancer”, and all articles were published in the 2017–2022 period. The systematic search yielded 230 studies from the initial search in PubMed, of which 125 articles met the inclusion criteria of reporting findings from breast cancer datasets using UK Biobank as a source (please refer to Supplementary Tables 1–10 for links to article references and data type). The other 105 records were removed as they did not meet the inclusion criteria or were not associated with research papers.

### 3.2 Analysis of genomic data

From the total of 125 papers included, 76 focused on genetic data available in the UK Biobank or other resources. These papers focussed on genetic variations such as single nucleotide polymorphisms (SNPs). From the total of 76 genetic data studies, 20 reported genetic variants related to breast cancer, meaning both prevalent and incident breast cancer, and 1 reported significant genes related to breast cancer. As some papers incorporated data from additional genome-wide association studies, these findings were also integrated in the analysis of genomic data. The excluded papers focused on genetic variants that were not directly associated with breast cancer. For example, they were investigating the causal relationship between a risk factor for breast cancer and the development of breast cancer, employing a method known as Mendelian randomization. All publications featuring genetics as data type, as well as their reason for exclusion and source for inclusion can be found in Supplementary Table 1 and 9.

These variants, all linked to both prevalent and incidence breast cancer risk, were merged together, and duplicates were removed until 2870 variants with reference SNP ID’s (rsID) remained. If rsIDs were not initially provided, Kaviar was utilised to identify and complete the missing rsIDs [30]. In this process, Kaviar utilised the reported chromosome and position information to generate all known rsIDs associated with that genetic position. The reference allele and alternate/effect allele provided in the study were then cross-checked against the genetic variant data given in the paper to complete and validate the rsID assignments. Next, to obtain rsID-related genes and pathways, SNPnexus was utilised (https://www.snp-nexus.org/v4/). SNPnexus is a web-based tool designed for the analysis of single nucleotide polymorphisms (SNPs) and their potential functional implications [31]. The 2870 variants were inserted, GRCh38/hg38 was selected and under ‘Annotation Categories’ Reactome was selected. Gene and pathway data were subsequently subtracted from the GeneProtein and Reactome data [32] provided via SNPnexus, respectively.

The GeneMANIA plug-in in Cytoscape software (v 3.9.1) was utilised to visualise the gene-gene interaction based on co-expression [33]. GeneMANIA serves as a valuable tool and database for predicting gene functions by leveraging a wide array of networks derived from diverse genomic and proteomic datasets [33]. We utilised cBioPortal, an open access resource for cancer genomics widely employed for investigating common gene alterations in various cancer types [34], [35], [36], to examine gene alteration levels in different subyptes of breast cancer. Initially, all breast cancer studies were selected, and the 'Pam50 + Claudin-low subtype' option was chosen from the 'Charts' dropdown menu. Subsequently, within the 'Pam50 + Claudin-low subtype' category, we further refined our search by selecting the following subtypes: Luminal A, Luminal B, HER2, Normal, and Basal. Mutated gene data were then downloaded for each subtype.The overall results were visualised using R (v 4.2.3) in R Studio (Build 421) and using Circos software package [37]. .

**Fig. 2:**
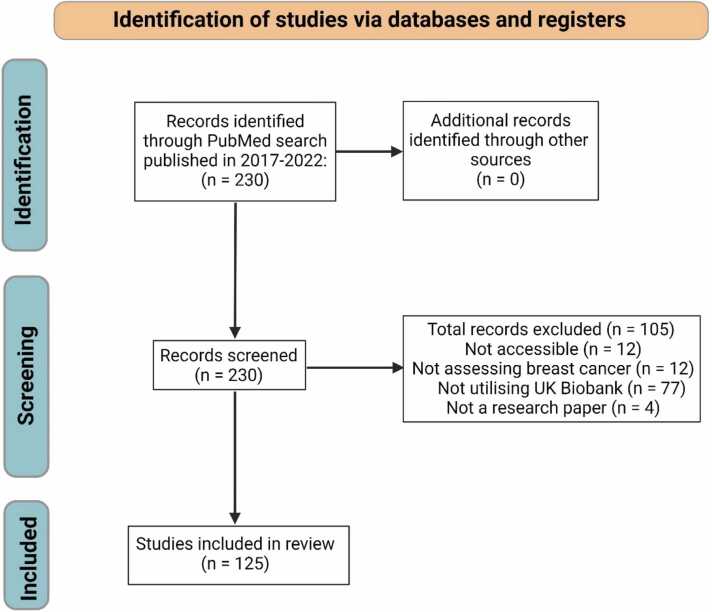
**PRISMA flowchart displaying the study selection process.** A total of 230 studies were identified from PubMed. Following the screening of titles and abstracts, 105 studies were excluded. A total of 125 articles that met the inclusion criteria were included.

**Fig. 3:**
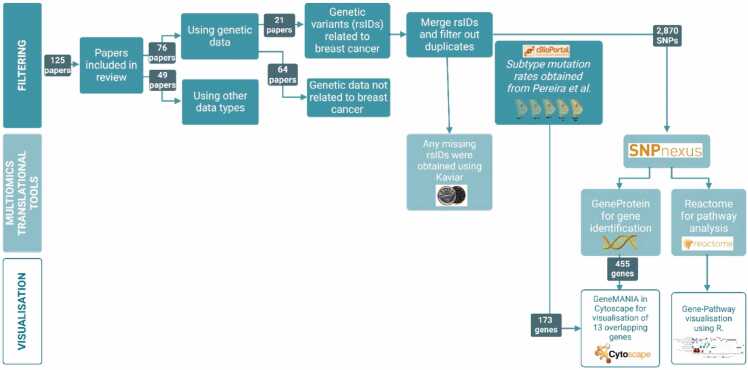
**Flowchart displaying the analysis of genetic data from the filtering process to the multiomics and visualisation tools used.** Further analysis was carried out in papers containing genetic data related to breast cancer. A final list of 2870 breast cancer related-rsIDs was run through SNPnexus and SNP-associated gene and pathways were visualised using Cytoscape (v 3.9.1) and R (v 4.2.3). Stratification by breast cancer subtype was analysed using cBioPortal, obtaining data from study by *Pereira et al*.

## 4 Results

### 4.1 Quantifying UK Biobank data-types utilised in breast cancer research.

**Fig. 4:**
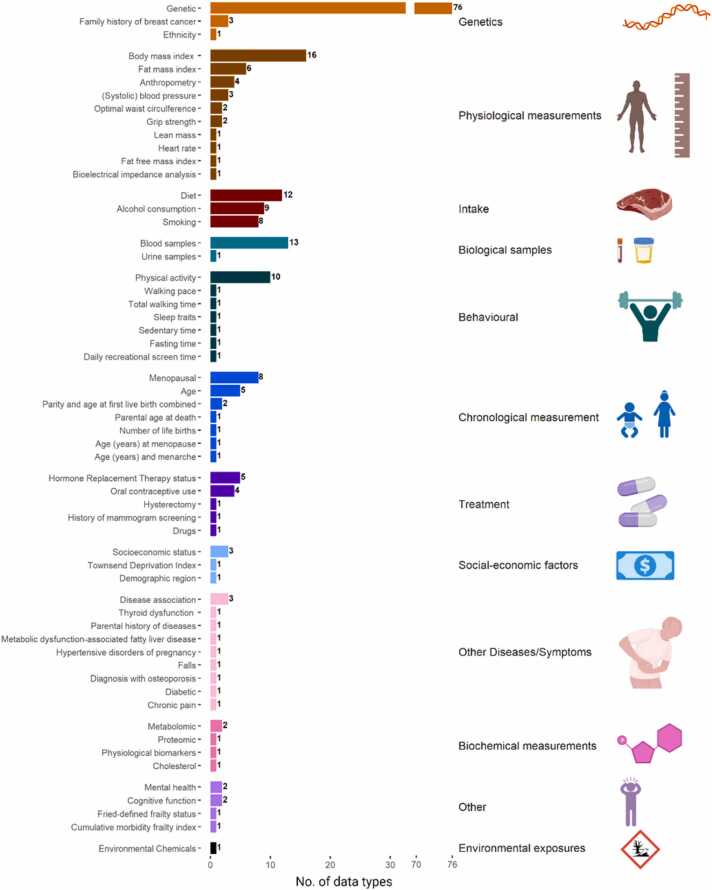
**Data types utilised in UK Biobank breast cancer studies.** All studies explored one or more datatypes classified in 12 categories. The 12 categories of data types encompass a wide array of information, ranging from demographic and clinical data to imaging and biomarker data. Among these data types, genetic data emerged as the most extensively investigated in breast cancer research. Aspects created with BioRender.com.

All 125 studies included UK Biobank data as either stand alone or in conjunction with other biobanks or databases. These studies focused on investigating breast cancer, with 25.6% (n = 32) specifically targeting breast cancer, 49.6% (n = 62) exploring multiple cancers, and 24.8% (n = 31) examining various pathologies. To test their hypotheses, all the studies utilised one or more data types. The predominant data type utilised across the publications was genetic data (60.8%), followed by body mass index (12.8%) and blood sample data (10.4%) – highlighted in Fig. 4. All 125 studies are referenced in the Supplementary Tables 1–10, categorized by their respective categories where applicable.

### 4.2 Linking of data types

**Fig. 5:**
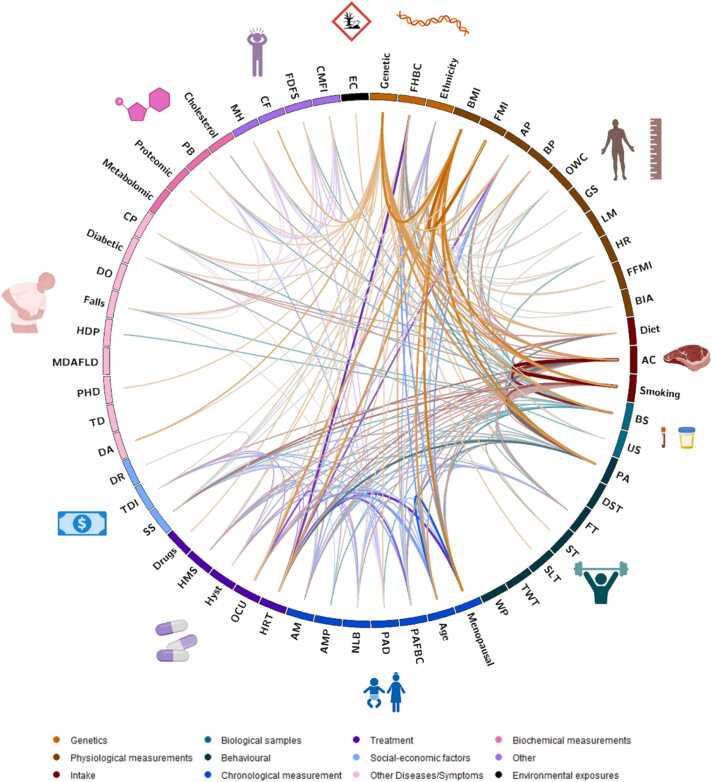
**Circos plot shows combinations of data types used in UK Biobank breast cancer research.** All included studies explored one or more datatype classified in 12 color-coded categories. The bidirectional connections between the data types are coloured according to data type which are most linked to other data types in general. For instance, as genetic data types are most linked, the link between ‘genetic’ and ‘proteomic’ is coloured orange and not pink. The colour-intensity of connections between data types indicates their degree of association within UK Biobank breast cancer publications. Genetic, BMI, smoking, alcohol consumption and physical activity data exhibit higher connectivity, while proteomic and metabolomic data show lower or no connections. Abbreviations clockwise: FHBC = Family History of Breast Cancer; BMI = Body Mass Index; FMI = Fat Mass Index; AP = Anthropometry; BP = Blood Pressure; OWC = Optimal Waist Circumference; GS = Grip Strength; LM = Lean Mass; HR = Heart rate; FFMI = Fat Free Mass Index; BIA = Bioelectrical Impedance Analysis; AC = Alcohol Consumption; BS = Blood Samples; US = Urine Samples; PA = Physical Activity; DST = Daily Screen Time; FT = Fasting Time; ST = Sedentary Time; SLT = Sleep Traits; TWT = Total Walking Time; WP = Walking Pace; PAFBC = Parity and Age at First live Birth Combined; PAD = Parental Age at Death; NLB = Number of Life Births; AMP = Age at Menopause; AM = Age at Menarche; HRT = Hormone Replacement Therapy; OCU = Oral Contraceptive Use; Hyst = Hysterectomy; HMS = History of Mammogram Screening; SS = Socioeconomic Status; TDI = Townsend Deprivation Index; DR = Demographic Region; DA = Disease Association; TD = Thyroid Dysfunction; PHD = Parental History of Diseases; MDAFLD = Metabolic Dysfunction-Associated Fatty Liver Disease; HDP = Hypertensive Disorders of Pregnancy; DO = Diagnosis with osteoporosis; CP = Chronic Pain; PB = Physiological Biomarkers; MH = Mental Health; CF = Cognitive Function; FDFS = Fried-Defined Frailty Status; CMFI = Cumulative Morbidity Frailty Index; EC = Environmental Chemicals. Aspects created with BioRender.com**.**

We examined the combinations made between the exploited data types in breast cancer research. The 125 included publications utilised a total of 58 datatypes. As shown through the cord diagram in Fig. 5 (created using the Circos software package - http://circos.ca/), certain data types are studied together with other datatypes more frequently, while other datatypes are used on their own. Notably, among the 58 different data types explored, 36 were used in a single study, encompassing drug use, environmental chemicals, and proteomic data.

### 4.3 Discovery analysis of genetic alteration stratified by breast cancer subtypes

Out of the 74 publications investigating genetic data related to breast cancer, 20 studies identified a collective total of 2870 unique breast cancer related rsIDs associated with breast cancer. Additionally, one study identified 10 significant genes linked to breast cancer. We investigated the relationship of the 2870 rsIDs to genes using SNPnexus, adding in the significant related genes of one study, resulting in 455 breast cancer related genes.

**Fig. 6:**
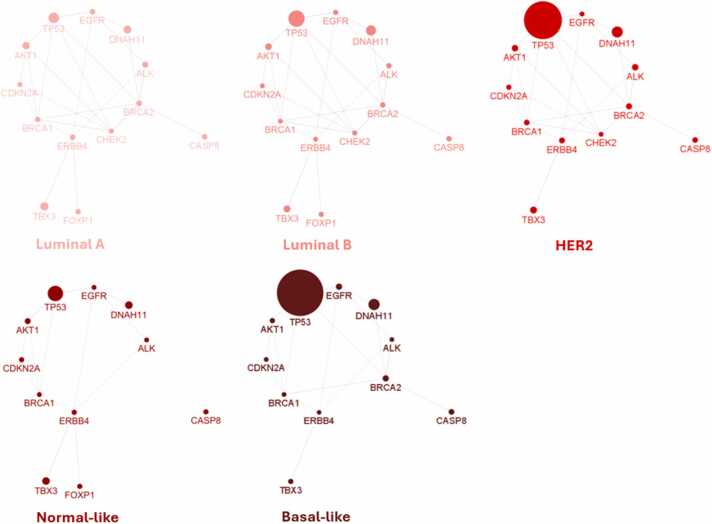
**13 breast cancer associated genes containing different rates of genetic alteration in breast cancer intrinsic subtypes.** The number of breast cancer tumors per subtype is as follows: luminal A (n = 679), luminal B (n = 461), HER2 (n = 220), normal-like (n = 140) and basal-like (n = 199). The intensity of the colour mirrors the proliferation rate, with darker shades representing higher rates. Node size corresponds to the percentage of mutated genes within specific subtypes. Edges illustrate co-expression relationships between genes.

Further analysis of genetic alterations among breast cancer intrinsic subtypes (Normal-like; Basal-like; Luminal-A; Luminal-B; HER-2) [38] revealed varying levels of alterations in a total of 13 genes, as depicted in Fig. 6. The mutation rates differed significantly across subtypes, with basal-like tumors exhibiting the highest overall mutation rate (11.32%), while luminal A (3.42%) and luminal B (4.25%) breast cancers displayed comparatively lower rates. Notably, tumour protein p53 (*TP53)* (43.50%) and dynein axonemal heavy chain 11 (*DNAH11)* (10.42%) emerged as the most frequently altered genes in breast cancer tumors, followed by T-box transcription factor 3 (*TBX3)* (4.98%), AKT serine/threonine kinase 1 (*AKT1)* (3.60%), and breast cancer gene 2 (*BRCA2)* (2.53%). Some breast cancer subtypes, appeared to have no mutation in certain genes: normal-like in *BRCA2* and forkhead box p1 (*FOXP1)* and basal like in checkpoint kinase 2 (*CHEK2)* and *FOXP1*. Supplementary Table 11 lists all 455 genes, along with their percentage mutation rates, average mutation rates per subtype and per gene.

### 4.4 Pathways potentially affected by genetic alteration

**Fig. 7:**
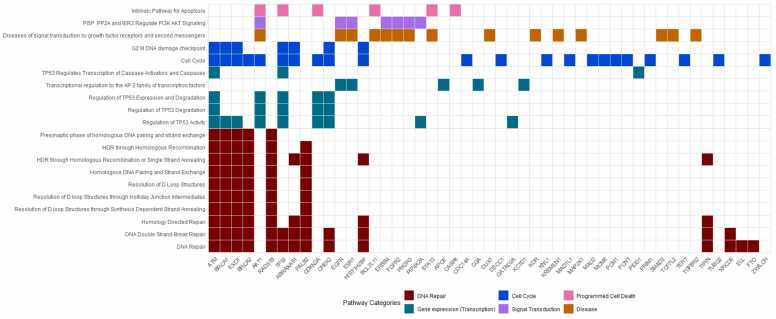
**Involvement of breast cancer-associated genes in biological pathways.** The top 20 most significant pathways (p < 0.001) containing at least one of the 10 differently mutated genes (*EGFR*, *TP53*, *BRCA2*, *CASP8*, *CHEK2*, *ERBB4*, *FOXP1*, *BRCA1*, *CDKN2A*, *AKT1*) established in former analysis. Pathways shown are color-coded based on pathway categories. Cells in the matrix indicate gene-pathway associations, with genes predominantly present in these pathways are positioned on the left, with their presence gradually decreasing towards the right (Pathway details, alongside rsID, p-value and gene associations are provided in Supplementary Table 12).

We then investigated the potential pathways affected by mutations by utilising the initial SNP pool of 2870 breast cancer SNPs derived from 21 publications. These SNPs were subsequently queried in SNPnexus using Reactome. The 20 most significant pathways containing the 10 breast cancer associated genes (*TP53,* epidermal growth factor receptor (*EGFR), BRCA2,* caspase 8 (*CASP8),* erb-b4 receptor tyrosine kinas 4 (*ERBB4), FOXP1,* breast cancer gene 1 *(BRCA1),* cyclin dependent kinase inhibitor 2a (*CDKN2A), AKT1, CHEK2*) were visualised in Fig. 7. Three genes (*DNAH11*, *TBX2*, ALK receptor tyrosine kinase (*ALK*)) were not included due to the absence of related pathways.

Among the 20 pathways, we observed that 50% were linked to DNA repair mechanisms, while 20% were associated with gene expression. Out of the 10 subtyped genes, *BRCA1* was found to be the most common across these pathways, followed by *BRCA2*, *AKT1* and *TP53*. *TP53* brought forward by its high difference in mutation rate among subtypes (Fig. 6), is present in 9 of the 20 significant pathways across 4 different pathway categories.

## 5 Discussion

Breast cancer remains the leading cause of cancer-related mortality for women globally. In this study, we investigated how UK Biobank data is utilised in breast cancer research. We have observed a lack of studies that have effectively combined these data types thus far. Our findings demonstrate that the majority studies published in the last 5 years have primarily focused on incorporating genetic data from breast cancer patients. The combination of breast cancer-related variants found in genetic data studies gave rise to a list of both well-known and unknown genes to be related to breast cancer. Different genetic alteration rates were found in these genes across subtype. These genes are mainly clustered in DNA repair and signal transduction pathways.

Over the past few years, there has been a significant increase in the utilisation UK Biobank data, evidenced by the rising number of approved proposals and published studies [39], [40]. The most frequently requested data type in the UK Biobank comprises of mortality, cancer and genetics corresponding to the common usage of genetic data in breast cancer studies as shown in Fig. 4.

Given the increasing utilisation of UK Biobank data and the prominent role of genetic data in breast cancer studies, it is notable that the UK Biobank provides a valuable resource, allowing for the integration of various omics approaches, including proteomic and metabolomic analysis, to further advance our understanding of breast cancer subtypes. This concept is evidenced by a recent study stating that combining UK Biobank genomics data to other omics data facilitates the dissection of the molecular underpinnings of disease [41]. Another study demonstrated the utilisation of multiomic data aiding the classification subtypes of endocrine hypertension [42]. Multiomic analysis has emerged as a powerful tool in breast cancer research, propelling advancements in areas such as subtype classification [41], therapeutic response [43] or to study the role of specific genes [44]. However, it is worth noting that the sample sizes of these studies have been relatively small; 487, 168 and 1109 cases, respectively. In this regard, the UK Biobank presents a significant opportunity for multiomics research, as breast cancer is the most common cancer diagnosed within the UK Biobank with a substantial cohort of 9117 prevalent and 8761 incident breast cancer patients [45].

As of the time of composing this review and the timeline of when these studies were analysed (2017–2022), there is a scarcity of published datasets and research focusing on the analysis of proteomics and metabolomics datasets. The limited utilisation of multiomic data in studying breast cancer could arise from several factors. Firstly, the delayed release of these specific datasets, with genetic data having been made available prior to the proteomic and metabolomic counterparts. Furthermore, the intricacies associated with integrating diverse datasets for comprehensive model development pose a significant challenge, contributing to the observed to the observed constraints in incorporating multi-omic approaches in breast cancer research.

Our investigation brings to light that a considerable number of studies draw upon genetic data sourced from the UK Biobank to explore breast cancer. Most cancers, including breast cancer are characterised by irregular and uncontrolled cellular growth caused in some degree by genomic instability. Genomic instability differs among intrinsic subtypes and manifests as mutations and is intrinsically associated with variations in patient survival, clinical outcome, the development of metastatic or recurrent tumors, and serves as a predictive indicator of tumor responsiveness to anti-cancer drugs [46], [47]. Our findings support the appearance of alterations in DNA repair pathway genes known to drive additional genetic mutations in breast cells. More specifically, SNPs in *BRCA1* and *BRCA2* genes are well-known risk factors for breast cancer in addition to other DNA repair genes such as *EXO1* and *CHEK2*. Additionally, we observed significant disparities in genetic alterations among the intrinsic subtypes of breast cancer, a trend not fully captured by the number of breast cancer-related SNPs identified in our analysis. For instance, despite BRCA1 and BRCA2 exhibiting lower mutation rates, the number of breast cancer-associated SNPs reported in literature is notably higher, particularly in comparison to TP53. Upon closer examination of the variants highlighted by the UK Biobank studies, it becomes apparent that rs78378222 and rs35850753 have been previously investigated as rare germline variants in neuroblastoma [48], however, no studies linking them to breast cancer have been identified.

Upon closer examination of the variants within these genes, certain variants such as rs11571833 and rs17879961, were previously associated with other cancer types, including lung cancer [49], urinary tract cancer [50] and pancreatic ductal adenocarcinoma [51]. However, rs11571833 and rs17879961 are located on well-established breast cancer susceptibility genes *BRCA2* and *CHECK2* respectively [52]. Alongside *BRCA2* and *CHECK2*, other well-known breast cancer-related genes involved in DNA repair mechanisms, such as *TP53*, *EXO1* and *PALB2* were identified in this study.

Our subtype-specific analysis and pathway analysis confirmed the involvement of all genes previously associated with breast cancer. However, it is important to note that this observation may be attributed to limitations within the utilized study providing mutation rates across different cancer subtypes. While our findings support existing knowledge regarding the role of these genes in breast cancer, further investigations are needed on the other genes we found to comprehensively assess their significance within specific subtypes and to address any potential biases inherent in the mutation rate data. However, no other studies report on this at this time point.

In conclusion, utilisation of the UK Biobank has not, until now, been effectively used to identify previously unknown breast cancer associated genes. However, the findings reveal the promising potential of harnessing multiomics approaches, or other combinations of data types using the extensive UK Biobank cohort to unravel the intricate cancer biology underlying distinct subtypes of breast cancer. This can contribute to enhancing breast cancer diagnosis, prognosis, the identification of biomarkers and precise treatment targets, thereby advancing personalized medicine in breast cancer care.

## Author Statement

LvdD designed the overall concepts of the article, performed data analysis and wrote the article. PK assisted in data analysis. AM provided insight in to study design and writing the paper. CHJ/JLS provide background on omics analysis in breast cancer. AL provided clinical background in breast cancer. ZR helped in study design and drafting of the article. NJWR conceptualised the overall work and wrote the article.

## CRediT authorship contribution statement

**Lisa van den Driest:** Conceptualization, Writing – original draft, Writing – review & editing. **Patricia Kelly:** Conceptualization, Methodology, Writing – original draft, Writing – review & editing. **Alan Marshall:** Conceptualization, Writing – original draft, Writing – review & editing. **Caroline H Johnson:** Conceptualization, Methodology, Writing – original draft, Writing – review & editing. **Jessica Lasky-Su:** Conceptualization, Investigation, Methodology, Writing – original draft, Writing – review & editing. **Alison Lannigan:** Conceptualization, Writing – original draft, Writing – review & editing. **Zahra Rattray:** Conceptualization, Methodology, Supervision, Writing – original draft, Writing – review & editing. **Nicholas J.W. Rattray:** Conceptualization, Data curation, Formal analysis, Funding acquisition, Investigation, Methodology, Project administration, Resources, Supervision, Validation, Writing – original draft, Writing – review & editing.

## Conflict Statement

Please take this letter as evidence that no authors declare any conflict of interest with any aspect of this research article.
