# Supplementary material for: A gap analysis of UK biobank publications reveals SNPs associated with intrinsic subtypes of breast cancer
Source: Comput Struct Biotechnol J. 2024 May 10;23:2200–10. doi: 10.1016/j.csbj.2024.05.001 (PMC11137368; doi:10.1016/j.csbj.2024.05.001)
Supplement: Supplementary file 1 — Supplementary material [file mmc1.docx]

A Gap Analysis of UK Biobank Publications revealing Breast Cancer associated SNPs interpreted into subtype specific gene analysis.

Lisa van den Driest^1^, Patricia Kelly^1^, Alan Marshall^2^, Caroline H Johnson^3^, Jessica Lasky-Su^4^, Alison Lannigan^5+6^, Zahra Rattray^1+5^ & Nicholas JW Rattray^1+5^

1. Strathclyde Institute of Pharmacy and Biomedical Sciences, University of Strathclyde, 161 Cathedral Street, Glasgow, UK, G4 0RE
2. School of Social and Political Science, University of Edinburgh, Chrystal Macmillan Building, George Square, Edinburgh, UK, EH8 9LD
3. Yale School of Public Health, Yale University, 60 College Street, New Haven, CT, USA, 06510
4. Brigham and Women's Hospital and Harvard Medical School, 181 Longwood Ave, Boston, MA 02115
5. NHS Lanarkshire, Lanarkshire, Scotland, UK.
6. Wishaw General Hospital, NHS Lanarkshire, Scotland, UK.

**Supplementary**

125 Included Studies ordered by categories

**Supplementary Table 1|** Publications utilizing ‘genetics’ data type. In total 12 studies utilised genetic data to predict breast cancer. *Some studies used other GWAS datasets such as: *Michailidou, K. et al.* comprised of 61,282 female cases of breast cancer and 45,494 female controls of European ancestry from 68 studies in the Breast Cancer Association Consortium. *Mavaddat, N. et al.* comprised of 94,075 case participants and 75,017 control participants of European ancestry from 69 studies in the Breast Cancer Association Consortium^1^.

| *Reference* | Data type | Inclusion | Reason for Inclusion/Exclusion as breast cancer genetic risk markers |
| --- | --- | --- | --- |
| *Ahmed, M. et al. Adiposity and cancer: a Mendelian randomization analysis in the UK biobank. Int J Obes (Lond) 45, 2657-2665, doi:10.1038/s41366-021-00942-y (2021).* | Genetic | No | Mendelian randomization: Genetic data reflect on high adiposity. |
| *Ahmed, M. et al. Considering hormone-sensitive cancers as a single disease in the UK biobank reveals shared aetiology. Commun Biol 5, 614, doi:10.1038/s42003-022-03554-y (2022).* | Genetic | No | SNPs used in this study is utilised to estimate heritability in hormone-sensitive cancers. No SNPs specifically related to breast cancer were reported. |
| *Alharbi, A. F. & Parrington, J. The role of genetic polymorphisms in endolysosomal ion channels TPC2 and P2RX4 in cancer pathogenesis, prognosis, and diagnosis: a genetic association in the UK Biobank. NPJ Genom Med 6, 58, doi:10.1038/s41525-021-00221-9 (2021).* | Genetic | Yes | 3 SNPs in this study were found to be associated with a higher general risk of developing breast cancer: rs35264875:TA, rs72932540:GA, rs28360472:GA. |
| *Au Yeung, S. L. & Schooling, C. M. Impact of glycemic traits, type 2 diabetes and metformin use on breast and prostate cancer risk: a Mendelian randomization study. BMJ Open Diabetes Res Care 7, e000872 (2019). https://doi.org:10.1136/bmjdrc-2019-000872* | Genetic | No | Mendelian randomisation: Genetic data associated with glycemic traits, type 2 diabetes. |
| *Au Yeung, S. L., Luo, S. & Schooling, C. M. The impact of GDF-15, a biomarker for metformin, on the risk of coronary artery disease, breast and colorectal cancer, and type 2 diabetes and metabolic traits: a Mendelian randomisation study. Diabetologia 62, 1638-1646, doi:10.1007/s00125-019-4913-2 (2019).* | Genetic | No* | This study obtained genetic data for breast cancer risk loci from *Michailidou, K. et al^2^.* Other genetic data utilised does not target breast cancer. |
| *Chan, II, Kwok, M. K. & Schooling, C. M. Blood pressure and risk of cancer: a Mendelian randomization study. BMC Cancer 21, 1338, doi:10.1186/s12885-021-09067-x (2021).* | Genetic | No | Mendelian randomisation: Genetic data associated with systolic and diastolic blood pressure, total- and site-specific cancers and asthma. |
| *Choi, J. et al. Associations of genetic susceptibility to 16 cancers with risk of breast cancer overall and by intrinsic subtypes. HGG Adv 3, 100077 (2022). https://doi.org:10.1016/j.xhgg.2021.100077* | Genetic | Yes | Study uses known susceptibility variants associated with breast cancer. SNPs in Supplementary tables 4 and 5. |
| *Clifton, L., Collister, J. A., Liu, X., Littlejohns, T. J. & Hunter, D. J. Assessing agreement between different polygenic risk scores in the UK Biobank. Sci Rep 12, 12812, doi:10.1038/s41598-022-17012-6 (2022).* | Genetic | No* | This study includes polygenic risk score from *Michailidou, K. et al^2^. and Mavaddat, N. et al^1^.* |
| *Fritsche, L. G. et al. On cross-ancestry cancer polygenic risk scores. PLoS Genet 17, e1009670 (2021). https://doi.org:10.1371/journal.pgen.1009670* | Genetic | No* | This study includes polygenic risk score from *Michailidou, K. et al^2^.* |
| *Ge, T., Chen, C. Y., Ni, Y., Feng, Y. A. & Smoller, J. W. Polygenic prediction via Bayesian regression and continuous shrinkage priors. Nat Commun 10, 1776, doi:10.1038/s41467-019-09718-5 (2019).* | Genetic | No* | This study includes polygenic risk score from *Michailidou, K. et al^2^.* |
| *Hassanin, E. et al. Breast and prostate cancer risk: The interplay of polygenic risk, rare pathogenic germline variants, and family history. Genet Med 24, 576-585 (2022). https://doi.org:10.1016/j.gim.2021.11.009* | Genetic | Yes | This study includes germline variants in known breast cancer genes (ATM, BRCA1, BRCA2, PALB2, CHEK2). |
| *Hu, J., Li, T., Wang, S. & Zhang, H. Supervariants identification for breast cancer. Genet Epidemiol 44, 934-947, doi:10.1002/gepi.22350 (2020).* | Genetic | Yes | Table 6. displays SNPs associated with breast cancer. |
| *Guochong, J. et al. Evaluating the Utility of Polygenic Risk Scores in Identifying High-Risk Individuals for Eight Common Cancers. JNCI Cancer Spectr 4, pkaa021, doi:10.1093/jncics/pkaa021 (2020).* | Genetic | Yes | Supplementary Table 1 contains GWAS-identified SNPs for PRS of each cancer including breast cancer. |
| *Johansson, Å. et al. Investigating the Effect of Estradiol Levels on the Risk of Breast, Endometrial, and Ovarian Cancer. J Endocr Soc 6, bvac100 (2022). https://doi.org:10.1210/jendso/bvac100* | Genetic | No | Mendelian randomisation: genetic variants associated with estradiol levels. |
| *Kramer, I. et al. Breast Cancer Polygenic Risk Score and Contralateral Breast Cancer Risk. Am J Hum Genet 107, 837-848, doi:10.1016/j.ajhg.2020.09.001 (2020).* | Genetic | No* | This study obtained genetic data for breast cancer risk loci from *Michailidou, K. et al.^2^ and Mavaddat, N. et al.^1^* |
| *Läll, K. et al. Polygenic prediction of breast cancer: comparison of genetic predictors and implications for risk stratification. BMC Cancer 19, 557 (2019). https://doi.org:10.1186/s12885-019-5783-1* | Genetic | Yes | This study compares genetic predictors across different cohorts, including |
| *Larsson, S. C. et al. Insulin-like growth factor-1 and site-specific cancers: A Mendelian randomization study. Cancer Med 9, 6836-6842 (2020). https://doi.org:10.1002/cam4.3345* | Genetic | No | Mendelian Randomisation: Genetic data associated with insulin-like growth factor-1 |
| *Larsson, S. C. et al. Genetically proxied milk consumption and risk of colorectal, bladder, breast, and prostate cancer: a two-sample Mendelian randomization study. BMC Med 18, 370, doi:10.1186/s12916-020-01839-9 (2020).* | Genetic | No* | This study obtained genetic data for breast cancer risk loci from *Michailidou, K. et al^2^.* |
| *Larsson, S. C. et al. Genetically predicted plasma phospholipid arachidonic acid concentrations and 10 site-specific cancers in UK biobank and genetic consortia participants: A mendelian randomization study. Clin Nutr 40, 3332-3337 (2021). https://doi.org:10.1016/j.clnu.2020.11.004* | Genetic | No | Mendelian randomisation: SNPs associated with plasma phospholipid arachidonic acid concentrations. |
| *Larsson, S. C., Lee, W. H., Kar, S., Burgess, S. & Allara, E. Assessing the role of cortisol in cancer: a wide-ranged Mendelian randomisation study. Br J Cancer 125, 1025-1029, doi:10.1038/s41416-021-01505-8 (2021).* | Genetic | No | Mendelian randomisation: SNPs associated with cortisol levels. |
| *Larsson, S. C. et al. Serum Estradiol and 20 Site-Specific Cancers in Women: Mendelian Randomization Study. J Clin Endocrinol Metab 107, e467-e474 (2022). https://doi.org:10.1210/clinem/dgab713* | Genetic | No | Mendelian randomisation: SNPs associated with endogenous E2 levels in cancer. |
| *Lello, L., Raben, T. G., Yong, S. Y., Tellier, L. & Hsu, S. D. H. Genomic Prediction of 16 Complex Disease Risks Including Heart Attack, Diabetes, Breast and Prostate Cancer. Sci Rep 9, 15286 (2019). https://doi.org:10.1038/s41598-019-51258-x* | Genetic | No | No genetic variants associated with breast cancer found in this study. |
| *Lello, L., Raben, T. G. & Hsu, S. D. H. Sibling validation of polygenic risk scores and complex trait prediction. Sci Rep 10, 13190, doi:10.1038/s41598-020-69927-7 (2020).* | Genetic | No | The study focusses on differences in disease risk between siblings and doesn’t specifically identifies genetic variants for breast cancer. |
| *Li, M., Kwok, M. K., Fong, S. S. M. & Schooling, C. M. Indoleamine 2,3-dioxygenase and ischemic heart disease: a Mendelian Randomization study. Sci Rep 9, 8491, doi:10.1038/s41598-019-44819-7 (2019).* | Genetic | No | Mendelian randomisation: genetic variants associated with IDO1 and KAT3 proteins were utilised in this study. |
| *Liu, L. et al. Using machine learning to identify gene interaction networks associated with breast cancer. BMC Cancer 22, 1070, doi:10.1186/s12885-022-10170-w (2022).* | Genetic | Yes | This study found 2 SNPs significantly associated with breast cancer risk: rs1137101 and rs4655555, both on *LEPR* gene. |
| *Lu, Y. et al. Assessment of causal effects of visceral adipose tissue on risk of cancers: a Mendelian randomization study. International Journal of Epidemiology 51, 1204-1218, doi:10.1093/ije/dyac025 (2022).* | Genetic | No | Mendelian randomisation: SNPs associated with visceral adipose tissue. |
| *Mavaddat, N. et al. Polygenic Risk Scores for Prediction of Breast Cancer and Breast Cancer Subtypes. The American Journal of Human Genetics 104, 21-34, doi:10.1016/j.ajhg.2018.11.002 (2019).* | Genetic | Yes | This study creates a polygenic risk score of breast cancer and breast cancer subtypes. Used UK Biobank and BCAC data. |
| *McManus, J. M., Vargas, R., Bazeley, P. S., Schumacher, F. R. & Sharifi, N. Association Between Adrenal-Restrictive HSD3B1 Inheritance and Hormone-Independent Subtypes of Endometrial and Breast Cancer. JNCI Cancer Spectr 6 (2022). https://doi.org:10.1093/jncics/pkac061* | Genetic | Yes | Genetic variant rs1047303 on gene *HSD3B1*, is associated with basal breast cancer subtype. |
| *Meisner, A., Kundu, P. & Chatterjee, N. Case-Only Analysis of Gene-Environment Interactions Using Polygenic Risk Scores. Am J Epidemiol 188, 2013-2020, doi:10.1093/aje/kwz175 (2019).* | Genetic | No* | This study obtained polygenic risk score for breast cancer risk from *Michailidou, K. et al.^2^* and applied it to the 151 SNPs available in the UK Biobank data. |
| *Morales Berstein, F. et al. Assessing the causal role of epigenetic clocks in the development of multiple cancers: a Mendelian randomization study. Elife 11, doi:10.7554/eLife.75374 (2022).* | Genetic | No | Mendelian randomization: SNPs associated with epigenetic age clocks. |
| *Nounu, A., Kar, S. P., Relton, C. L. & Richmond, R. C. Sex steroid hormones and risk of breast cancer: a two-sample Mendelian randomization study. Breast Cancer Res 24, 66 (2022). https://doi.org:10.1186/s13058-022-01553-9* | Genetic | No | Mendalian randomization: genetic variants associated with sex steroid hormones. |
| *Ong, J. S. et al. Vitamin D and overall cancer risk and cancer mortality: a Mendelian randomization study. Hum Mol Genet 27, 4315-4322, doi:10.1093/hmg/ddy307 (2018).* | Genetic | No | Mendalian randomization: genetic variants associated with circulating 25(OH)D concentration. |
| *Pang, S. et al. Genetic and modifiable risk factors combine multiplicatively in common disease. Clin Res Cardiol 112, 247-257, doi:10.1007/s00392-022-02081-4 (2023).* | Genetic | No* | This study obtained risk variants for breast cancer risk from *Michailidou, K. et al.^2^* |
| *Patel, A. P. et al. Association of Rare Pathogenic DNA Variants for Familial Hypercholesterolemia, Hereditary Breast and Ovarian Cancer Syndrome, and Lynch Syndrome With Disease Risk in Adults According to Family History. JAMA Netw Open 3, e203959 (2020). https://doi.org:10.1001/jamanetworkopen.2020.3959* | Genetic | Yes | Supplementary Table 2. Risk alleles on *BRCA1* and *BRCA2* classified as pathogenic in breast cancer identified. ClinVar/Variation ID was translated using (https://www.ncbi.nlm.nih.gov/clinvar/variation/) |
| *Privé, F., Aschard, H. & Blum, M. G. B. Efficient Implementation of Penalized Regression for Genetic Risk Prediction. Genetics 212, 65-74, doi:10.1534/genetics.119.302019 (2019).* | Genetic | No | This study applies polygenic risk scores and compares its performance with other strategies. No specific breast cancer associated-SNPs are utilised in this study. |
| *Reinbolt, R. E. et al. Genomic risk prediction of aromatase inhibitor-related arthralgia in patients with breast cancer using a novel machine-learning algorithm. Cancer Med 7, 240-253, doi:10.1002/cam4.1256 (2018).* | Genetic | Yes | This study presents some SNPs and related genes associated with breast cancer pathophysiology |
| *Seyed Khoei, N. et al. Genetically Raised Circulating Bilirubin Levels and Risk of Ten Cancers: A Mendelian Randomization Study. Cells 10, doi:10.3390/cells10020394 (2021).* | Genetic | No | Mendelian randomization: The SNPs were associated with circulating total bilirubin levels |
| *Sun, X. et al. Identification of significant genes and therapeutic agents for breast cancer by integrated genomics. Bioengineered 12, 2140-2154, doi:10.1080/21655979.2021.1931642 (2021).* | Genetic | Yes | This study identifies 10 significant genes (*CLDN7, MLLT10, RBM33, SH3RF1, SSBP4, UBE2Z, BMPER, FGF7, MSRB3, and TNRC6B*) |
| *Tang, S. N., Zuber, V. & Tsilidis, K. K. Identifying and ranking causal biochemical biomarkers for breast cancer: a Mendelian randomisation study. BMC Med 20, 457 (2022). https://doi.org:10.1186/s12916-022-02660-2* | Genetic | No | Mendelian randomization: The SNPs were associated with biochemical biomarkers. |
| *Thompson, D. J. et al. Genetic predisposition to mosaic Y chromosome loss in blood. Nature 575, 652-657, doi:10.1038/s41586-019-1765-3 (2019).* | Genetic | Yes | Supplementary Table 14 shows the overlap between mLOY and cancer associated loci, including breast cancer. |
| *Wang, F. et al. Genome-Wide Analysis of Rare Haplotypes Associated with Breast Cancer Risk. Cancer Res 83, 332-345, doi:10.1158/0008-5472.Can-22-1888 (2023).* | Genetic | Yes | Table 2. Identification of Thirteen Rare Haplotype Loci for Breast Cancer Risk in the UK Biobank Phased Data |
| *Walsh, K. M. et al. Pleiotropic MLLT10 variation confers risk of meningioma and estrogen-mediated cancers. Neurooncol Adv 4, vdac044 (2022). https://doi.org:10.1093/noajnl/vdac044* | Genetic | Yes | This study reports one SNP rs7084454, as a risk factor for ER+ breast cancer, but protective against ER- breast cancer. |
| *Weedon, M. N. et al. Use of SNP chips to detect rare pathogenic variants: retrospective, population based diagnostic evaluation. Bmj 372, n214 (2021). https://doi.org:10.1136/bmj.n214* | Genetic | No | This study aims to verify the sensitivity and specificity of SNP chips are sufficient for the detection of rare pathogenic variants. No specific variants for breast cancer are reported. |
| *Wu, J. & Tan, X. The role of MTNR1B polymorphism on circadian rhythm-related cancer: A UK Biobank cohort study. Int J Cancer 151, 888-896, doi:10.1002/ijc.34047 (2022).* | Genetic | No | The SNPs are associated with chronotype. |
| *Jiang, X., Ge, T. & Chen, C. Y. The causal role of circulating vitamin D concentrations in human complex traits and diseases: a large-scale Mendelian randomization study. Sci Rep 11, 184, doi:10.1038/s41598-020-80655-w (2021).* | Genetic | No | Mendelian randomization: The SNPs were associated with circulating 25(OH)D concentration. |
| *Xin, J. et al. Association between circulating vitamin E and ten common cancers: evidence from large-scale Mendelian randomization analysis and a longitudinal cohort study. BMC Med 20, 168, doi:10.1186/s12916-022-02366-5 (2022).* | Genetic | No | Mendelian randomization: The SNPs were associated with circulating vitamin E. |
| *Yong, S. Y., Raben, T. G., Lello, L. & Hsu, S. D. H. Genetic architecture of complex traits and disease risk predictors. Sci Rep 10, 12055, doi:10.1038/s41598-020-68881-8 (2020).* | Genetic | Yes | Supplementary Table 26 shows list of genes responsible for predicting breast cancer. Supplementary Table 49 gives a list of breast cancer predictor SNPs located on FGFR2 and TOX3 genes. |
| *Yuan, S. et al. Effects of tumour necrosis factor on cardiovascular disease and cancer: A two-sample Mendelian randomization study. EBioMedicine 59, 102956 (2020). https://doi.org:10.1016/j.ebiom.2020.102956* | Genetic | No | Mendelian randomization: The SNPs were associated with tumour necrosis factor levels. |
| *Yuan, S. et al. Causal associations of thyroid function and dysfunction with overall, breast and thyroid cancer: A two-sample Mendelian randomization study. Int J Cancer 147, 1895-1903, doi:10.1002/ijc.32988 (2020).* | Genetic | No | Mendelian randomization: The SNPs were associated with thyroid function and dysfunction. |
| *Yuan, S. et al. Iron Status and Cancer Risk in UK Biobank: A Two-Sample Mendelian Randomization Study. Nutrients 12, doi:10.3390/nu12020526 (2020).* | Genetic | No | Mendelian randomization: The SNPs were associated with iron status. |
| *Yuan, S. et al. Is Type 2 Diabetes Causally Associated With Cancer Risk? Evidence From a Two-Sample Mendelian Randomization Study. Diabetes 69, 1588-1596, doi:10.2337/db20-0084 (2020).* | Genetic | No | Mendelian randomization: The SNPs were associated with type 2 diabetes, fasting glucose, fasting insulin. |
| *Zhang, Y. et al. Bone mineral density and risk of breast cancer: A cohort study and Mendelian randomization analysis. Cancer 128, 2768-2776, doi:10.1002/cncr.34252 (2022).* | Genetic | No | Mendelian randomization: The SNPs were associated with bone mineral density. |

**Supplementary Table 2|** Publications utilizing ‘Physiological measurements’ data type.

| Reference | Data type |
| --- | --- |
| *Celis-Morales, C. A. et al. Associations of grip strength with cardiovascular, respiratory, and cancer outcomes and all cause mortality: prospective cohort study of half a million UK Biobank participants. Bmj 361, k1651, doi:10.1136/bmj.k1651 (2018).* | Grip strength |
| *Parra-Soto, S. et al. Combined association of general and central obesity with incidence and mortality of cancers in 22 sites. Am J Clin Nutr 113, 401-409, doi:10.1093/ajcn/nqaa335 (2021).* | Optimal waist circumference |
| *Peila, R., Arthur, R. S., Dannenberg, A. J. & Rohan, T. E. Association of a Healthy Lifestyle Index with Risk of Breast Cancer among Women with Normal Body Mass Index in the UK Biobank. Cancer Epidemiol Biomarkers Prev 31, 554-560, doi:10.1158/1055-9965.Epi-21-0765 (2022).* | Body mass index |
| *Parra-Soto, S., Ho, F. K., Pell, J. P. & Celis-Morales, C. Does insulin-like growth factor moderate the association between height and risk of cancer at 24 sites? Br J Cancer 123, 1697-1704, doi:10.1038/s41416-020-01059-1 (2020).* | Height |

**Supplementary Table 3|** Publications utilizing ‘Intake’ data type.

| Reference | Data type |
| --- | --- |
| Anderson, J. J. *et al.* Red and processed meat consumption and breast cancer: UK Biobank cohort study and meta-analysis. *European Journal of Cancer* 90, 73-82, doi:10.1016/j.ejca.2017.11.022 (2018). | Diet |
| Deng, Y., Ge, W., Xu, H. & Zhang, J. A Mendelian randomization study of the effect of tea intake on breast cancer. *Front Nutr* 9, 956969 (2022). https://doi.org:10.3389/fnut.2022.956969 | Diet |
| Karavasiloglou, N., Pestoni, G., Kühn, T. & Rohrmann, S. Adherence to cancer prevention recommendations and risk of breast cancer in situ in the United Kingdom Biobank. *Int J Cancer* 151, 1674-1683, doi:10.1002/ijc.34183 (2022). | Diet |
| Liu, Z. *et al.* Association between fish oil supplementation and cancer risk according to fatty fish consumption: A large prospective population-based cohort study using UK Biobank. *Int J Cancer* 150, 562-571, doi:10.1002/ijc.33819 (2022). | Diet |
| Ong, J. S. *et al.* Association between coffee consumption and overall risk of being diagnosed with or dying from cancer among >300 000 UK Biobank participants in a large-scale Mendelian randomization study. *Int J Epidemiol* 48, 1447-1456, doi:10.1093/ije/dyz144 (2019). | Diet |
| Parra-Soto, S. *et al.* Association of meat, vegetarian, pescatarian and fish-poultry diets with risk of 19 cancer sites and all cancer: findings from the UK Biobank prospective cohort study and meta-analysis. *BMC Med* 20, 79, doi:10.1186/s12916-022-02257-9 (2022). | Diet |
| Watling, C. Z. *et al.* Risk of cancer in regular and low meat-eaters, fish-eaters, and vegetarians: a prospective analysis of UK Biobank participants. *BMC Med* 20, 73, doi:10.1186/s12916-022-02256-w (2022). | Diet |
| Yaghjyan, L., Rich, S., Mao, L., Mai, V. & Egan, K. M. Interactions of coffee consumption and postmenopausal hormone use in relation to breast cancer risk in UK Biobank. *Cancer Causes Control* 29, 519-525, doi:10.1007/s10552-018-1028-x (2018). | Diet |
|  |  |
| Association of meat, vegetarian, pescatarian and fish-poultry diets with risk of 19 cancer sites and all cancer: findings from the UK Biobank prospective cohort study and meta-analysis | Diet |

**Supplementary Table 4|** Publications utilizing ‘Biological samples’ data type.

| Reference | Data type |
| --- | --- |
| *Knuppel, A. et al. Circulating Insulin-like Growth Factor-I Concentrations and Risk of 30 Cancers: Prospective Analyses in UK Biobank. Cancer Res 80, 4014-4021 (2020). https://doi.org:10.1158/0008-5472.Can-20-1281* | Blood samples |
| *Murphy, N. et al. Insulin-like growth factor-1, insulin-like growth factor-binding protein-3, and breast cancer risk: observational and Mendelian randomization analyses with ∼430 000 women. Ann Oncol 31, 641-649 (2020). https://doi.org:10.1016/j.annonc.2020.01.066* | Blood samples |
| *Rentsch, C. T. et al. Risk of 16 cancers across the full glycemic spectrum: a population-based cohort study using the UK Biobank. BMJ Open Diabetes Res Care 8 (2020). https://doi.org:10.1136/bmjdrc-2020-001600* | Blood samples |
| *Tin Tin, S., Reeves, G. K. & Key, T. J. Endogenous hormones and risk of invasive breast cancer in pre- and post-menopausal women: findings from the UK Biobank. Br J Cancer 125, 126-134, doi:10.1038/s41416-021-01392-z (2021).* | Blood samples |
| *Qian, F. & Huo, D. Circulating Insulin-Like Growth Factor-1 and Risk of Total and 19 Site-Specific Cancers: Cohort Study Analyses from the UK Biobank. Cancer Epidemiol Biomarkers Prev 29, 2332-2342, doi:10.1158/1055-9965.Epi-20-0743 (2020).* | Blood samples |

**Supplementary Table 5|** Publications utilizing ‘Behavioral’ data type.

| Reference | Data type |
| --- | --- |
| Celis-Morales, C. A. *et al.* Walking Pace Is Associated with Lower Risk of All-Cause and Cause-Specific Mortality. *Med Sci Sports Exerc* 51, 472-480, doi:10.1249/mss.0000000000001795 (2019). | Walking pace, Total walking time |
| Guo, W., Fensom, G. K., Reeves, G. K. & Key, T. J. Physical activity and breast cancer risk: results from the UK Biobank prospective cohort. *Br J Cancer* 122, 726-732, doi:10.1038/s41416-019-0700-6 (2020). | Physical activity |
| Hunter, R. F., Murray, J. M. & Coleman, H. G. The association between recreational screen time and cancer risk: findings from the UK Biobank, a large prospective cohort study. *Int J Behav Nutr Phys Act* 17, 97 (2020). https://doi.org:10.1186/s12966-020-00997-6 | Daily recreational screening time |
| Murray, J. M., Coleman, H. G. & Hunter, R. F. Physical activity and cancer risk: Findings from the UK Biobank, a large prospective cohort study. *Cancer Epidemiol* 68, 101780, doi:10.1016/j.canep.2020.101780 (2020). | Physical activity |

**Supplementary Table 6|** Publications utilizing ‘Treatment’ data type.

| Reference | Data type |
| --- | --- |
| Karlsson, T., Johansson, T., Höglund, J., Ek, W. E. & Johansson, Å. Time-Dependent Effects of Oral Contraceptive Use on Breast, Ovarian, and Endometrial Cancers. *Cancer Res* 81, 1153-1162, doi:10.1158/0008-5472.Can-20-2476 (2021). | Oral contraceptive use |

**Supplementary Table 7|** Publications utilizing ‘Other disease/symptoms’ data type.

| Reference | Data type |
| --- | --- |
| Foster, M. & Niedzwiedz, C. L. Associations between multimorbidity and depression among breast cancer survivors within the UK Biobank cohort: a cross-sectional study. *BMC Cancer* 21, 650, doi:10.1186/s12885-021-08409-z (2021). | Depression |
| Liu, Z. *et al.* Metabolic dysfunction-associated fatty liver disease and the risk of 24 specific cancers. *Metabolism* 127, 154955 (2022). https://doi.org:10.1016/j.metabol.2021.154955 | Metabolic dysfunction-associated fatty liver disease |
| Rodríguez-Gómez, I. *et al.* Osteoporosis and Its Association With Cardiovascular Disease, Respiratory Disease, and Cancer: Findings From the UK Biobank Prospective Cohort Study. *Mayo Clin Proc* 97, 110-121, doi:10.1016/j.mayocp.2021.07.019 (2022). | Diagnosis with osteoporosis |
| Tran, T. V. *et al.* Thyroid dysfunction and breast cancer risk among women in the UK Biobank cohort. *Cancer Med* 10, 4604-4614, doi:10.1002/cam4.3978 (2021). | Thyroid dysfunction |

**Supplementary Table 8|** Publications utilizing ‘Biochemical measurements’ data type.

| Reference | Data type |
| --- | --- |
| Buergel, T. *et al.* Metabolomic profiles predict individual multidisease outcomes. *Nat Med* 28, 2309-2320, doi:10.1038/s41591-022-01980-3 (2022). | Metabolic |

**Supplementary Table 9|** Publications utilizing ‘Other’ data type.

| Reference | Data type |
| --- | --- |
| Niedzwiedz, C. L., Robb, K. A., Katikireddi, S. V., Pell, J. P. & Smith, D. J. Depressive symptoms, neuroticism, and participation in breast and cervical cancer screening: Cross-sectional and prospective evidence from UK Biobank. *Psychooncology* 29, 381-388, doi:10.1002/pon.5272 (2020). | Mental health |

**Supplementary Table 10|** Publications utilizing ‘Combinations of data types’ data type. For publications including ‘genetic’ data type, inclusion and reason for inclusion is specified.

| Reference | Data type | Inclusion | Reason for Inclusion/Exclusion |
| --- | --- | --- | --- |
| *Al-Ajmi, K., Lophatananon, A., Ollier, W. & Muir, K. R. Risk of breast cancer in the UK biobank female cohort and its relationship to anthropometric and reproductive factors. PLoS One 13, e0201097, doi:10.1371/journal.pone.0201097 (2018).* | Family history of breast cancer, Anthropometry, Body mass index, Age, Menopausal, Oral contraceptive use, HRT status |  |  |
| *Al Ajmi, K., Lophatananon, A., Mekli, K., Ollier, W. & Muir, K. R. Association of Nongenetic Factors With Breast Cancer Risk in Genetically Predisposed Groups of Women in the UK Biobank Cohort. JAMA Netw Open 3, e203760, doi:10.1001/jamanetworkopen.2020.3760 (2020).* | **Genetic**, Body weight, Physical activity, Alcohol consumption, Oral contraceptive use, HRT status | No* | This study obtained the polygenic risk score for breast cancer risk loci from *Mavaddat, N. et al.^1^* |
| *Amin, H. A. et al. Mendelian randomisation analyses of UK Biobank and published data suggest that increased adiposity lowers risk of breast and prostate cancer. Sci Rep 12, 909, doi:10.1038/s41598-021-04401-6 (2022).* | Body mass index, Fat mass index |  |  |
| *Arthur, R. S., Dannenberg, A. J., Kim, M. & Rohan, T. E. The association of body fat composition with risk of breast, endometrial, ovarian and colorectal cancers among normal weight participants in the UK Biobank. Br J Cancer 124, 1592-1605 (2021). https://doi.org:10.1038/s41416-020-01210-y* | Body mass index, Fat mass index |  |  |
| *Arthur, R. S., Dannenberg, A. J. & Rohan, T. E. The association of prediagnostic circulating levels of cardiometabolic markers, testosterone and sex hormone-binding globulin with risk of breast cancer among normal weight postmenopausal women in the UK Biobank. Int J Cancer 149, 42-57, doi:10.1002/ijc.33508 (2021).* | Blood samples, Socioeconomic status, Ethnicity, Fasting time, Age (years) and menarche, HRT status, Age (years) at menopause, Family history of breast cancer, Physical activity, Alcohol consumption, Smoking, Parity and age at first live birth combined, History of mammogram screening |  |  |
| *Arthur, R. S., Wang, T., Xue, X., Kamensky, V. & Rohan, T. E. Genetic Factors, Adherence to Healthy Lifestyle Behavior, and Risk of Invasive Breast Cancer Among Women in the UK Biobank. JNCI: Journal of the National Cancer Institute 112, 893-901, doi:10.1093/jnci/djz241 (2020).* | **Genetic**, Diet, Alcohol consumption, Anthropometry, Physical activity | Yes | Supplementary Table 2 contains SNPs included in PRS score for prediction of breast cancer created by *Mavaddat, N. et al.* Polygenic Risk Scores for Prediction of Breast Cancer and Breast Cancer Subtypes. *American journal of human genetics 104(1):21-34 doi:10.1016/j.ajhg.2018.11.002 (2019)* |
| *Cao, Z. et al. Association of obesity status and metabolic syndrome with site-specific cancers: a population-based cohort study. Br J Cancer 123, 1336-1344, doi:10.1038/s41416-020-1012-6 (2020).* | Disease association, Body mass index |  |  |
| *Dixon-Suen, S. C. et al. Physical activity, sedentary time and breast cancer risk: a Mendelian randomisation study. Br J Sports Med 56, 1157-1170 (2022). https://doi.org:10.1136/bjsports-2021-105132* | **Genetic**, Physical activity, Sedentary time | No | Mendelian randomization: The SNPs were associated with wrist-worn accelerometer-measured overall physical activity, sedentary time, accelerometer-measured or self-reported vigorous physical activity. |
| *Elwood, P. C. et al. Healthy living and cancer: evidence from UK Biobank. Ecancermedicalscience 12, 792, doi:10.3332/ecancer.2018.792 (2018).* | Body mass index (BMI), Height, Body weight, Smoking, Alcohol consumption, Physical activity, Diet |  |  |
| *Fang, X. et al. Causal association of childhood obesity with cancer risk in adulthood: A Mendelian randomization study. Int J Cancer 149, 1421-1425 (2021). https://doi.org:10.1002/ijc.33691* | **Genetic**, Proteomic | Yes | Table 5 consists of 22 protein-associated SNPs associated with breast cancer risk |
| *Gong, L. et al. Integrative, genome-wide association study identifies chemicals associated with common women's malignancies. Genomics 112, 5029-5036 (2020). https://doi.org:10.1016/j.ygeno.2020.09.011* | **Genetic**, Environmental Chemicals | Yes | This study identifies chemicals associated with breast cancer. |
| *Guo, W., Key, T. J. & Reeves, G. K. Adiposity and breast cancer risk in postmenopausal women: Results from the UK Biobank prospective cohort. Int J Cancer 143, 1037-1046, doi:10.1002/ijc.31394 (2018).* | Body weight, Body mass index, Fat mass index, Menopausal |  |  |
| *Harrison, S. et al. The causal effects of health conditions and risk factors on social and socioeconomic outcomes: Mendelian randomization in UK Biobank. Int J Epidemiol 49, 1661-1681, doi:10.1093/ije/dyaa114 (2020).* | Alcohol consumption, Body mass index, Cholesterol, Systolic blood pressure, Smoking, Socioeconomic status |  |  |
| *He, Q. et al. Association of body composition with risk of overall and site-specific cancers: A population-based prospective cohort study. Int J Cancer 149, 1435-1447, doi:10.1002/ijc.33697 (2021).* | Bioelectrical impedance analysis, Lean mass, Fat mass index |  |  |
| *Jiang, X. et al. Uncovering variable neoplasms between ATM protein-truncating and common missense variants using 394 694 UK Biobank exomes. Genes Chromosomes Cancer 61, 523-529, doi:10.1002/gcc.23042 (2022).* | Disease association, Genetic |  |  |
| *Jin, C. et al. Association between dried fruit intake and pan-cancers incidence risk: A two-sample Mendelian randomization study. Front Nutr 9, 899137 (2022). https://doi.org:10.3389/fnut.2022.899137* | Diet, **Genetic** | No | Mendelian randomization: The SNPs were associated with dried fruit intake. |
| *Karlsson, T., Johansson, T., Höglund, J., Ek, W. E. & Johansson, Å. Time-Dependent Effects of Oral Contraceptive Use on Breast, Ovarian, and Endometrial Cancers. Cancer Res 81, 1153-1162, doi:10.1158/0008-5472.Can-20-2476 (2021).* | Oral contraceptive use, Year of birth, Hysterectomy, Menopausal, Townsend Deprivation Index, Body mass index, Smoking, Number of life births |  |  |
| *Khan, Z. et al. Genetic variation associated with thyroid autoimmunity shapes the systemic immune response to PD-1 checkpoint blockade. Nat Commun 12, 3355, doi:10.1038/s41467-021-23661-4 (2021).* | Genetic,  **Disease** association | No | Polygenic risk score is constructed based on lifetime risk of hypothyroidism. High PRS is found to increase the risk of atezolizumab-induced thyroid dysfunction and lower risk of death in triple negative breast cancer. |
| *Kuo, C. L. et al. The Longevity-Associated SH2B3 (LNK) Genetic Variant: Selected Aging Phenotypes in 379,758 Subjects. J Gerontol A Biol Sci Med Sci 75, 1656-1662, doi:10.1093/gerona/glz191 (2020).* | **Genetic**, Cognitive function, Blood samples, Parental age at death, Mental health, Chronic pain, Falls, cumulative morbidity frailty index, Fried-defined frailty status, Physiological biomarkers | Yes | rs3184504 was associated with breast cancer risk |
| *Lai, F. Y. et al. Adult height and risk of 50 diseases: a combined epidemiological and genetic analysis. BMC Med 16, 187, doi:10.1186/s12916-018-1175-7 (2018).* | **Genetic**, Height | No | Mendelian randomization: The SNPs were associated with height. |
| *Larsson, S. C. et al. Smoking, alcohol consumption, and cancer: A mendelian randomisation study in UK Biobank and international genetic consortia participants. PLoS Med 17, e1003178, doi:10.1371/journal.pmed.1003178 (2020).* | **Genetic**, Smoking, Alcohol consumption | No | Mendelian randomization: The SNPs were associated with smoking initiation, alcohol consumption. |
| *Lehrer, S. & Rheinstein, P. H. Breast Cancer, Alzheimer's Disease, and APOE4 Allele in the UK Biobank Cohort. J Alzheimers Dis Rep 5, 49-53, doi:10.3233/adr-200266 (2021).* | **Genetic**, Cognitive function | No | SNP is associated with apolipoprotein E4 (*APOE4*), associated with cognitive decline in breast cancer patients, not breast cancer directly. |
| *Lu, T., Forgetta, V., Richards, J. B. & Greenwood, C. M. T. Polygenic risk score as a possible tool for identifying familial monogenic causes of complex diseases. Genet Med 24, 1545-1555, doi:10.1016/j.gim.2022.03.022 (2022).* | **Genetic**, Parental history of diseases | Yes | Supplementary Table 2 and 3 contain SNPs associated with breast cancer. PALB2-associated SNPs for hereditary breast cancer. |
| *Magnus, M. C., Borges, M. C., Fraser, A. & Lawlor, D. A. Identifying potential causal effects of age at menopause: a Mendelian randomization phenome-wide association study. Eur J Epidemiol 37, 971-982, doi:10.1007/s10654-022-00903-3 (2022).* | Menopausal, Height |  |  |
| *Marderstein, A. R. et al. A polygenic-score-based approach for identification of gene-drug interactions stratifying breast cancer risk. Am J Hum Genet 108, 1752-1764 (2021). https://doi.org:10.1016/j.ajhg.2021.07.008* | **Genetic**, Drugs | No* | This study obtained genetic data for breast cancer risk loci from *Michailidou, K. et al.^2^ and Mavaddat, N. et al.^1^* |
| *Mason, K. E., Pearce, N. & Cummins, S. Neighborhood environment and socioeconomic inequalities in cancer admissions: a prospective study using UK Biobank and linked hospital records. Cancer Causes Control 33, 1431-1444 (2022). https://doi.org:10.1007/s10552-022-01626-2* | Socioeconomic status, Demographic region |  |  |
| *Papadimitriou, N. et al. Physical activity and risks of breast and colorectal cancer: a Mendelian randomisation analysis. Nat Commun 11, 597, doi:10.1038/s41467-020-14389-8 (2020).* | **Genetic**, Physical activity | No | Mendelian randomization: The SNPs were associated with physical activity. |
| *Park, H. A. et al. Mendelian randomisation study of smoking exposure in relation to breast cancer risk. Br J Cancer 125, 1135-1145, doi:10.1038/s41416-021-01432-8 (2021).* | **Genetic**, Smoking | No | Mendelian randomization: The SNPs were associated with smoking. |
| *Parra-Soto, S., Pell, J. P., Celis-Morales, C. & Ho, F. K. Absolute and relative grip strength as predictors of cancer: prospective cohort study of 445 552 participants in UK Biobank. J Cachexia Sarcopenia Muscle 13, 325-332, doi:10.1002/jcsm.12863 (2022).* | Grip strength, Body weight, Body mass index, Fat mass index |  |  |
| *Parra-Soto, S. et al. Associations of six adiposity-related markers with incidence and mortality from 24 cancers-findings from the UK Biobank prospective cohort study. BMC Med 19, 7 (2021). https://doi.org:10.1186/s12916-020-01848-8* | Body mass index, Height |  |  |
| *Peila, R., Arthur, R. & Rohan, T. E. Risk factors for ductal carcinoma in situ of the breast in the UK Biobank cohort study. Cancer Epidemiol 64, 101648, doi:10.1016/j.canep.2019.101648 (2020).* | Age, Height, Physical activity, Family history of breast cancer, Menopausal, Parity and age at first live birth combined, Anthropometry, Body mass index (BMI), HRT status |  |  |
| *Powell, M. J., Dufault, S. M., Gunderson, E. P. & Benz, C. C. Cancer and Cardiovascular Risk in Women With Hypertensive Disorders of Pregnancy Carrying a Common IGF1R Variant. Mayo Clin Proc 95, 2684-2696, doi:10.1016/j.mayocp.2020.03.037 (2020).* | Hypertensive disorders of pregnancy,  Blood samples |  |  |
| *Richmond, R. C. et al. Investigating causal relations between sleep traits and risk of breast cancer in women: mendelian randomisation study. Bmj 365, l2327, doi:10.1136/bmj.l2327 (2019).* | **Genetic**, Sleep traits | No | Mendelian randomization: The SNPs were associated with physical activity. |
| *Richardson, T. G., Sanderson, E., Elsworth, B., Tilling, K. & Davey Smith, G. Use of genetic variation to separate the effects of early and later life adiposity on disease risk: mendelian randomisation study. Bmj 369, m1203, doi:10.1136/bmj.m1203 (2020).* | **Genetic**, Body mass index | No | Mendelian randomization: The SNPs were associated with early and later life disease risk. |
| *Ruth, K. S. et al. Using human genetics to understand the disease impacts of testosterone in men and women. Nat Med 26, 252-258, doi:10.1038/s41591-020-0751-5 (2020).* | **Genetic**, Blood samples |  | Mendelian randomization: The SNPs were associated with sex hormone measures. |
| *Tin Tin, S., Key, T. J. & Reeves, G. K. Alcohol Intake and Endogenous Hormones in Pre- and Postmenopausal Women: Findings from the UK Biobank. Cancer Epidemiol Biomarkers Prev 30, 2294-2301 (2021). https://doi.org:10.1158/1055-9965.Epi-21-0789* | Blood samples, Alcohol consumption, Menopausal |  |  |
| *Vithayathil, M. et al. Body size and composition and risk of site-specific cancers in the UK Biobank and large international consortia: A mendelian randomisation study. PLoS Med 18, e1003706, doi:10.1371/journal.pmed.1003706 (2021).* | Height, Fat free mass index (FFMI), Fat mass index (FMI), Body mass index (BMI), **Genetic** | No | Mendelian randomization: The SNPs were associated with body size. |
| *Watts, E. L. et al. Prospective analyses of testosterone and sex hormone-binding globulin with the risk of 19 types of cancer in men and postmenopausal women in UK Biobank. Int J Cancer 149, 573-584, doi:10.1002/ijc.33555 (2021).* | Menopausal, Blood samples |  |  |
| *Wu, K. et al. The relationship between processed meat, red meat, and risk of types of cancer: A Mendelian randomization study. Front Nutr 9, 942155, doi:10.3389/fnut.2022.942155 (2022).* | **Genetic**, Diet | No | Mendelian randomization: The SNPs were associated with meat consumption. |
| *Xu, Z. et al. Menopause, hysterectomy, menopausal hormone therapy and cause-specific mortality: cohort study of UK Biobank participants. Hum Reprod 37, 2175-2185 (2022). https://doi.org:10.1093/humrep/deac137* | Menopausal, HRT status |  |  |
| *Yang, H., Pawitan, Y., Fang, F., Czene, K. & Ye, W. Biomarkers and Disease Trajectories Influencing Women's Health: Results from the UK Biobank Cohort. Phenomics 2, 184-193, doi:10.1007/s43657-022-00054-1 (2022).* | Urine samples, Blood samples, Body mass index,  Optimal waist circumference, Blood pressure, Heart rate |  |  |
| *Zhang, Y. B. et al. Associations of combined healthy lifestyles with cancer morbidity and mortality among individuals with diabetes: results from five cohort studies in the USA, the UK and China. Diabetologia 65, 2044-2055, doi:10.1007/s00125-022-05754-x (2022).* | Diabetic, Smoking, Alcohol consumption, Physical activity, Diet, Body mass index |  |  |
| *Zhu, M. et al. C-reactive protein and cancer risk: a pan-cancer study of prospective cohort and Mendelian randomization analysis. BMC Med 20, 301, doi:10.1186/s12916-022-02506-x (2022).* | **Genetic**, Blood samples | No | Mendelian randomization: The SNPs were associated with C-reactive protein. |

**Supplementary Table 11|** Breast cancer associated SNPs give rise to a list of 455 genes. To understand the differences between intrinsic subtypes and prevalent mutations in breast cancer, we utilised the cBioPortal platform, yielding one pertinent study by *Pereira et al*^3^. 13 genes were found to be differentially mutated across the different subtypes. The BC-associated SNPs are not specific for any intrinsic subtype. The other 442 genes found: *TNRC6B, SH2B3, MSRB3, FGF7, BMPER, UBE2Z, SSBP4, SH3RF1, RBM33, CLDN7, SLC28A1, PTPRK, FBXL17, RORA, ATM, PALB2, KLHDC7A, PEX14, HIVEP3, AC119676.1, NASP, GPBP1L1, TMEM69, MAST2, PIK3R3, AL358075.4, AL358075.1, AL358075.3, AL358075.2, POMGNT1, URAP1, NSUN4, FAF1, PKN2-AS1, CDC14A, GSTM4, AP4B1, AP4B1-AS1, DCLRE1B, AL157902.1, RF00561, PHGDH, EMBP1, POLR3C, MTMR11, CTXND2, KRTCAP2, AL713999.1, TRIM46, MUC1, THBS3, AC234582.1, GBAP1, PKLR, ASH1L, DAP3P1, DNM3, PHLDA3, LGR6, ZC3H11A, ZBED6, CD34, ESRRG, EXO1, FAM208B, FRMD4A, MLLT10, PIP4K2A, KIAA1217, PARD3, AL117339.5, ZNF365, AC024598.1, AC067752.1, AL450311.2, ZMIZ1, CEP55, TCF7L2, FGFR2, ANO9, PANO1, PIDD1, RPLP2, SNORA52, PNPLA2, LSP1, CREB3L1, AC090559.1, MIR4487, OVOL1, AP002989.1, C11orf65, KDELC2, ALG9, AP001781.2, ALG9-IT1, HTR3B, SIK3, APOA1-AS, CLMP, ST3GAL4, AP003500.1, AC007406.3, ATF7IP, CCDC91, HSD17B6, PRIM1, KCNMB4, PGAM1P5, CUX2, ATXN2, SH2B3, RNU4-2, SIRT4, RNU6-1088P, PLA2G1B, MSI1, RPS27P25, AC003982.1, FRY, EPSTI1, PAX9, AL079303.1, SLC25A21, RAD51B, CCDC88C, RIN3, ADSSL1, SIVA1.*

| Genes | Luminal A [n=679] | Luminal B [n=461] | HER2 [n=220] | Basal-like [n=199] | Normal-like [n=140] | Average mutation rate per gene | Breast cancer-associated SNPs |
| --- | --- | --- | --- | --- | --- | --- | --- |
| Average mutation rate per subtype | 3.42% | 4.25% | 9.18% | 11.32% | 4.21% |  |  |
| *TP53* | 11.9 % | 24.3% | 70.0% | 88.4% | 22.9% | 43.50% | rs78378222, rs35850753 |
| *TBX3* | 7.2% | 4.8% | 5.0% | 1.5% | 6.4% | 4.98% | rs1061657 |
| *DNAH11* | 6.5% | 11.5% | 13.6% | 14.1% | 6.4% | 10.42% | rs7785157, rs57104699, rs11381392, rs6948632, rs75341503 |
| *AKT1* | 5.4% | 4.1% | 4.1% | 1.5% | 2.9% | 3.60% | rs2494734, rs2498794 |
| *ALK* | 2.8% | 1.3% | 4.1% | 3.0% | 0.7% | 2.38% | rs4322799 |
| *BRCA2* | 1.9% | 1.5% | 3.2% | 3.5% | N/A | 2.53% | rs201523522, rs397507320, rs397507713, rs397507762, rs80359511, rs80358810, rs80359604, rs80359659, rs786204273, rs75096777, rs397515635, rs80359272, rs80359277, rs80359281, rs397507582, rs80359282, rs80359283, rs80359308, rs80359312, rs80359315, rs80359316, rs80359328, rs80358504, rs80358523, rs398122749, rs80359340, rs80359351, rs80358533, rs80359356, rs80358556, rs41293477, rs80359371, rs80359383, rs80358579, rs876659258, rs80359388, rs80359391, rs80359395, rs80359401, rs397507686, rs80358620, rs80359405, rs398122772, rs747070579, rs80359438, rs80359444, rs397507331, rs397507333, rs80359461, rs80359460, rs80359462, rs80358710, rs80359470, rs80358721, rs80359479, rs80359480, rs80359484, rs80359487, rs80359496, rs80359503, rs80359505, rs80359507, rs587782240, rs80359512, rs80359520, rs41293497, rs80358824, rs80359550, rs398122546, rs80358843, rs11571658, rs80359596, rs80359598, rs80359601, rs80359603, rs397507868, rs431825343, rs80359623, rs80359629, rs879255306, rs28897743, rs80359636, rs80359646, rs80358972, rs80358981, rs80359003, rs80359679, rs397507943, rs80359022, rs80359031, rs41293511, rs41293513, rs80359064, rs80359705, rs398122602, rs80359075, rs80359076, rs80359099, rs397507404, rs80359718, rs80359140, rs80359730, rs80359144, rs80359743, rs397508034, rs80359159, rs28897756, rs45580035, rs80359753, rs80359200, rs28897758, rs80359212, rs8035976, rs80359230, rs80359775, rs11571833 |
| *ERBB4* | 1.8% | 0.7% | 2.7% | 1.0% | 1.4% | 1.52% | rs190740846 |
| *EGFR* | 1.6% | 1.1% | 0.9% | 3.5% | 0.7% | 1.56% | rs13244925 |
| *CASP8* | 1.5% | 1.3% | 2.3% | 2.5% | 1.4% | 1.80% | rs3769823 |
| *BRCA1* | 1.3% | 2.0% | 2.3% | 5.0% | 0.7% | 2.26% | rs41293465, rs397509267, rs41293463, rs80357906, rs45553935, rs273901751, rs41293461, rs80358094,  rs45444999, rs80357243, rs28897696, rs41293459,  rs55770810, rs80357760, rs397507239, rs730882166,  rs80357542, rs80357813, rs80356885, rs41293455,  rs80357572, rs80357508, rs80357868, rs80357687,  rs80357729, rs62625308, rs80357018, rs80357573,  rs80356978, rs80357971, rs80357968, rs80357970,  rs80357960, rs80357426, rs80357853, rs80357522,  rs80357516, rs80357723, rs80356898, rs80357888,  rs62625303, rs80357939, rs80356991, rs273899695,  rs587782173, rs28897672  rs80356929 |
| *FOXP1* | 1.3% | 1.1% | N/A | N/A | 1.4% | 1.27% | rs148029062, rs13060421, rs13075282, rs11708920,  rs11706279, rs35967223, rs35540156, rs36023390,  rs9819066, rs56310245, rs62247160, rs61458295,  rs17108, rs34264449, rs9836630, rs9854068, rs9859717, rs9828629, rs9853081, rs35483362, rs11715828, rs6805189, rs62247166, rs13327339, rs9878602, rs7623765, rs11710593, rs13071807, rs34068332, rs11391200, rs9876924, rs9825432, rs3068915, rs2116709, rs878173, rs878172, rs9817016, rs6789491, rs9828619, rs2322031, rs369875440 |
| *CHEK2* | 0.9% | 0.9% | 0.5% | N/A | N/A | 0.77% | rs200432447, rs774175654, rs142763740, rs730881700,  rs137853011, rs587780174, rs200928781, rs555607708,  rs760502479, rs786203053, rs587782471, rs786202497,  rs587782245, rs796389290, rs72552322, rs17879961,  rs137853007, rs730881701, rs730881699, rs28909982,  rs587781269, rs587782070, rs141568342, rs761494650,  rs536907995, rs5997390 |
| *CDKN2A* | 0.3% | 0.7% | 1.4% | 0.5% | 1.4% | 0.86% | rs2811710 |

**Supplementary Table 12**| Pathway analysis

| Pathway | Pathway Category | Genes | rsID | P-value |
| --- | --- | --- | --- | --- |
| Resolution of D-loop Structures through Synthesis-Dependent Strand Annealing (SDSA) | DNA Repair | *ATM,BRCA1,BRCA2,EXO1,PALB2,RAD51B* | rs11571658,rs11571833,rs11624333,rs118203998,rs118203999,rs121434219,rs1800057,rs1801516,rs180177083,rs180177099,rs180177100,rs180177110,rs180177132,rs180177133,rs180177142,rs180177143,rs201523522,rs201963507,rs202206540,rs2588809,rs273899695,rs273901751,rs28897672,rs28897696,rs28897743,rs28897756,rs28897758,rs28904921,rs371638537,rs376603775,rs397507239,rs397507320,rs397507331,rs397507333,rs397507404,rs397507582,rs397507686,rs397507713,rs397507762,rs397507868,rs397507943,rs397508034,rs397509267,rs397515635,rs398122546,rs398122602,rs398122749,rs398122772,rs41293455,rs41293459,rs41293461,rs41293463,rs41293465,rs41293477,rs41293497,rs41293511,rs41293513,rs431825343,rs45444999,rs45476495,rs45553935,rs45580035,rs515726117,rs532480170,rs55770810,rs564652222,rs587776408,rs587776410,rs587776411,rs587776416,rs587776527,rs587779815,rs587779818,rs587779836,rs587779844,rs587779846,rs587779865,rs587779869,rs587779872,rs587780210,rs587781347,rs587781598,rs587781658,rs587781730,rs587781776,rs587781905,rs587782005,rs587782081,rs587782173,rs587782198,rs587782240,rs587782292,rs587782652,rs587782680,rs587782719,rs62625303,rs62625308,rs72755295,rs730881304,rs730881348,rs730881359,rs730881391,rs730881394,rs730882166,rs747070579,rs747727055,rs748634900,rs749036865,rs75096777,rs758588019,rs762083530,rs763470424,rs764925691,rs769523686,rs770641163,rs772821016,rs776294034,rs778269655,rs779278389,rs780905851,rs786201675,rs786201689,rs786201693,rs786201957,rs786202239,rs786203008,rs786203054,rs786203272,rs786203370,rs786203488,rs786203606,rs786203714,rs786204273,rs786204433,rs786204751,rs80356885,rs80356898,rs80356929,rs80356978,rs80356991,rs80357018,rs80357243,rs80357426,rs80357508,rs80357516,rs80357522,rs80357542,rs80357572,rs80357573,rs80357687,rs80357723,rs80357729,rs80357760,rs80357813,rs80357853,rs80357868,rs80357888,rs80357906,rs80357939,rs80357960,rs80357968,rs80357970,rs80357971,rs80358094,rs80358504,rs80358523,rs80358533,rs80358556,rs80358579,rs80358620,rs80358710,rs80358721,rs80358810,rs80358824,rs80358843,rs80358972,rs80358981,rs80359003,rs80359022,rs80359031,rs80359064,rs80359075,rs80359076,rs80359099,rs80359140,rs80359144,rs80359159,rs80359200,rs80359212,rs80359230,rs80359272,rs80359277,rs80359281,rs80359282,rs80359283,rs80359308,rs80359312,rs80359315,rs80359316,rs80359328,rs80359340,rs80359351,rs80359356,rs80359371,rs80359383,rs80359388,rs80359391,rs80359395,rs80359401,rs80359405,rs80359438,rs80359444,rs80359460,rs80359461,rs80359462,rs80359470,rs80359479,rs80359480,rs80359484,rs80359487,rs80359496,rs80359503,rs80359505,rs80359507,rs80359511,rs80359512,rs80359520,rs80359550,rs80359596,rs80359598,rs80359601,rs80359603,rs80359604,rs80359623,rs80359629,rs80359636,rs80359646,rs80359659,rs80359679,rs80359705,rs80359718,rs80359730,rs80359743,rs80359753,rs80359763,rs80359775,rs863225466,rs864622389,rs864622649,rs876659258,rs879255306 | 0.000002 |
| DNA Double-Strand Break Repair | DNA Repair | *ABRAXAS1,ATM,BRCA1,BRCA2,CHEK2,EXO1,HIST1H2BF,PALB2,RAD51B,TIPIN,TP53,XRCC6* | rs10008742,rs10012248,rs10015939,rs10016216,rs10023702,rs10025004,rs10027007,rs10032585,rs10033600,rs10213169,rs11099601,rs11571658,rs11571833,rs11624333,rs11727406,rs11728733,rs118203998,rs118203999,rs11930653,rs11935455,rs11940256,rs11947695,rs121434219,rs12649417,rs13112255,rs13121249,rs13128266,rs13128503,rs13129676,rs13139169,rs13142756,rs137853007,rs137853011,rs141568342,rs142763740,rs143767430,rs1565909,rs17352824,rs17879961,rs1800057,rs1801516,rs180177083,rs180177099,rs180177100,rs180177110,rs180177132,rs180177133,rs180177142,rs180177143,rs1964010,rs1980271,rs200432447,rs200928781,rs201523522,rs201963507,rs202206540,rs2132096,rs2132097,rs2172809,rs2177034,rs2588809,rs273899695,rs273901751,rs28488925,rs28603076,rs28653564,rs28783127,rs28785823,rs28869016,rs28869596,rs28875243,rs28895925,rs28897672,rs28897696,rs28897743,rs28897756,rs28897758,rs28904921,rs28909982,rs34136446,rs34803094,rs34961555,rs35000866,rs35452111,rs35608460,rs35850753,rs36014997,rs371638537,rs376603775,rs397507239,rs397507320,rs397507331,rs397507333,rs397507404,rs397507582,rs397507686,rs397507713,rs397507762,rs397507868,rs397507943,rs397508034,rs397509267,rs397515635,rs398122546,rs398122602,rs398122749,rs398122772,rs41293455,rs41293459,rs41293461,rs41293463,rs41293465,rs41293477,rs41293497,rs41293511,rs41293513,rs431825343,rs45444999,rs45476495,rs45553935,rs45580035,rs4693090,rs515726117,rs526064,rs532480170,rs536907995,rs538078,rs543318,rs555348,rs555607708,rs55770810,rs562723,rs564652222,rs568978,rs587776408,rs587776410,rs587776411,rs587776416,rs587776527,rs587779815,rs587779818,rs587779836,rs587779844,rs587779846,rs587779865,rs587779869,rs587779872,rs587780174,rs587780210,rs587781269,rs587781347,rs587781598,rs587781658,rs587781730,rs587781776,rs587781905,rs587782005,rs587782070,rs587782081,rs587782173,rs587782198,rs587782240,rs587782245,rs587782292,rs587782471,rs587782652,rs587782680,rs587782719,rs59644543,rs5997390,rs62625303,rs62625308,rs6535478,rs6834006,rs6838225,rs6844460,rs72552322,rs72755295,rs730881304,rs730881348,rs730881359,rs730881391,rs730881394,rs730881699,rs730881700,rs730881701,rs730882166,rs73161324,rs747070579,rs747727055,rs748634900,rs749036865,rs75096777,rs758588019,rs760502479,rs761494650,rs762083530,rs763470424,rs764925691,rs7663427,rs7666601,rs7671387,rs7691492,rs7694435,rs769523686,rs770641163,rs772821016,rs774175654,rs776294034,rs778269655,rs779278389,rs780905851,rs78378222,rs786201675,rs786201689,rs786201693,rs786201957,rs786202239,rs786202497,rs786203008,rs786203053,rs786203054,rs786203272,rs786203370,rs786203488,rs786203606,rs786203714,rs786204273,rs786204433,rs786204751,rs796389290,rs80356885,rs80356898,rs80356929,rs80356978,rs80356991,rs80357018,rs80357243,rs80357426,rs80357508,rs80357516,rs80357522,rs80357542,rs80357572,rs80357573,rs80357687,rs80357723,rs80357729,rs80357760,rs80357813,rs80357853,rs80357868,rs80357888,rs80357906,rs80357939,rs80357960,rs80357968,rs80357970,rs80357971,rs80358094,rs80358504,rs80358523,rs80358533,rs80358556,rs80358579,rs80358620,rs80358710,rs80358721,rs80358810,rs80358824,rs80358843,rs80358972,rs80358981,rs80359003,rs80359022,rs80359031,rs80359064,rs80359075,rs80359076,rs80359099,rs80359140,rs80359144,rs80359159,rs80359200,rs80359212,rs80359230,rs80359272,rs80359277,rs80359281,rs80359282,rs80359283,rs80359308,rs80359312,rs80359315,rs80359316,rs80359328,rs80359340,rs80359351,rs80359356,rs80359371,rs80359383,rs80359388,rs80359391,rs80359395,rs80359401,rs80359405,rs80359438,rs80359444,rs80359460,rs80359461,rs80359462,rs80359470,rs80359479,rs80359480,rs80359484,rs80359487,rs80359496,rs80359503,rs80359505,rs80359507,rs80359511,rs80359512,rs80359520,rs80359550,rs80359596,rs80359598,rs80359601,rs80359603,rs80359604,rs80359623,rs80359629,rs80359636,rs80359646,rs80359659,rs80359679,rs80359705,rs80359718,rs80359730,rs80359743,rs80359753,rs80359763,rs80359775,rs8042593,rs813298,rs863225466,rs864622389,rs864622649,rs876659258,rs879255306,rs9284657,rs9683689,rs971539,rs9997925 | 0.000008 |
| Resolution of D-loop Structures through Holliday Junction Intermediates | DNA Repair | *ATM,BRCA1,BRCA2,EXO1,PALB2,RAD51B* | rs11571658,rs11571833,rs11624333,rs118203998,rs118203999,rs121434219,rs1800057,rs1801516,rs180177083,rs180177099,rs180177100,rs180177110,rs180177132,rs180177133,rs180177142,rs180177143,rs201523522,rs201963507,rs202206540,rs2588809,rs273899695,rs273901751,rs28897672,rs28897696,rs28897743,rs28897756,rs28897758,rs28904921,rs371638537,rs376603775,rs397507239,rs397507320,rs397507331,rs397507333,rs397507404,rs397507582,rs397507686,rs397507713,rs397507762,rs397507868,rs397507943,rs397508034,rs397509267,rs397515635,rs398122546,rs398122602,rs398122749,rs398122772,rs41293455,rs41293459,rs41293461,rs41293463,rs41293465,rs41293477,rs41293497,rs41293511,rs41293513,rs431825343,rs45444999,rs45476495,rs45553935,rs45580035,rs515726117,rs532480170,rs55770810,rs564652222,rs587776408,rs587776410,rs587776411,rs587776416,rs587776527,rs587779815,rs587779818,rs587779836,rs587779844,rs587779846,rs587779865,rs587779869,rs587779872,rs587780210,rs587781347,rs587781598,rs587781658,rs587781730,rs587781776,rs587781905,rs587782005,rs587782081,rs587782173,rs587782198,rs587782240,rs587782292,rs587782652,rs587782680,rs587782719,rs62625303,rs62625308,rs72755295,rs730881304,rs730881348,rs730881359,rs730881391,rs730881394,rs730882166,rs747070579,rs747727055,rs748634900,rs749036865,rs75096777,rs758588019,rs762083530,rs763470424,rs764925691,rs769523686,rs770641163,rs772821016,rs776294034,rs778269655,rs779278389,rs780905851,rs786201675,rs786201689,rs786201693,rs786201957,rs786202239,rs786203008,rs786203054,rs786203272,rs786203370,rs786203488,rs786203606,rs786203714,rs786204273,rs786204433,rs786204751,rs80356885,rs80356898,rs80356929,rs80356978,rs80356991,rs80357018,rs80357243,rs80357426,rs80357508,rs80357516,rs80357522,rs80357542,rs80357572,rs80357573,rs80357687,rs80357723,rs80357729,rs80357760,rs80357813,rs80357853,rs80357868,rs80357888,rs80357906,rs80357939,rs80357960,rs80357968,rs80357970,rs80357971,rs80358094,rs80358504,rs80358523,rs80358533,rs80358556,rs80358579,rs80358620,rs80358710,rs80358721,rs80358810,rs80358824,rs80358843,rs80358972,rs80358981,rs80359003,rs80359022,rs80359031,rs80359064,rs80359075,rs80359076,rs80359099,rs80359140,rs80359144,rs80359159,rs80359200,rs80359212,rs80359230,rs80359272,rs80359277,rs80359281,rs80359282,rs80359283,rs80359308,rs80359312,rs80359315,rs80359316,rs80359328,rs80359340,rs80359351,rs80359356,rs80359371,rs80359383,rs80359388,rs80359391,rs80359395,rs80359401,rs80359405,rs80359438,rs80359444,rs80359460,rs80359461,rs80359462,rs80359470,rs80359479,rs80359480,rs80359484,rs80359487,rs80359496,rs80359503,rs80359505,rs80359507,rs80359511,rs80359512,rs80359520,rs80359550,rs80359596,rs80359598,rs80359601,rs80359603,rs80359604,rs80359623,rs80359629,rs80359636,rs80359646,rs80359659,rs80359679,rs80359705,rs80359718,rs80359730,rs80359743,rs80359753,rs80359763,rs80359775,rs863225466,rs864622389,rs864622649,rs876659258,rs879255306 | 0.000009 |
| Resolution of D-Loop Structures | DNA Repair | *ATM,BRCA1,BRCA2,EXO1,PALB2,RAD51B* | rs11571658,rs11571833,rs11624333,rs118203998,rs118203999,rs121434219,rs1800057,rs1801516,rs180177083,rs180177099,rs180177100,rs180177110,rs180177132,rs180177133,rs180177142,rs180177143,rs201523522,rs201963507,rs202206540,rs2588809,rs273899695,rs273901751,rs28897672,rs28897696,rs28897743,rs28897756,rs28897758,rs28904921,rs371638537,rs376603775,rs397507239,rs397507320,rs397507331,rs397507333,rs397507404,rs397507582,rs397507686,rs397507713,rs397507762,rs397507868,rs397507943,rs397508034,rs397509267,rs397515635,rs398122546,rs398122602,rs398122749,rs398122772,rs41293455,rs41293459,rs41293461,rs41293463,rs41293465,rs41293477,rs41293497,rs41293511,rs41293513,rs431825343,rs45444999,rs45476495,rs45553935,rs45580035,rs515726117,rs532480170,rs55770810,rs564652222,rs587776408,rs587776410,rs587776411,rs587776416,rs587776527,rs587779815,rs587779818,rs587779836,rs587779844,rs587779846,rs587779865,rs587779869,rs587779872,rs587780210,rs587781347,rs587781598,rs587781658,rs587781730,rs587781776,rs587781905,rs587782005,rs587782081,rs587782173,rs587782198,rs587782240,rs587782292,rs587782652,rs587782680,rs587782719,rs62625303,rs62625308,rs72755295,rs730881304,rs730881348,rs730881359,rs730881391,rs730881394,rs730882166,rs747070579,rs747727055,rs748634900,rs749036865,rs75096777,rs758588019,rs762083530,rs763470424,rs764925691,rs769523686,rs770641163,rs772821016,rs776294034,rs778269655,rs779278389,rs780905851,rs786201675,rs786201689,rs786201693,rs786201957,rs786202239,rs786203008,rs786203054,rs786203272,rs786203370,rs786203488,rs786203606,rs786203714,rs786204273,rs786204433,rs786204751,rs80356885,rs80356898,rs80356929,rs80356978,rs80356991,rs80357018,rs80357243,rs80357426,rs80357508,rs80357516,rs80357522,rs80357542,rs80357572,rs80357573,rs80357687,rs80357723,rs80357729,rs80357760,rs80357813,rs80357853,rs80357868,rs80357888,rs80357906,rs80357939,rs80357960,rs80357968,rs80357970,rs80357971,rs80358094,rs80358504,rs80358523,rs80358533,rs80358556,rs80358579,rs80358620,rs80358710,rs80358721,rs80358810,rs80358824,rs80358843,rs80358972,rs80358981,rs80359003,rs80359022,rs80359031,rs80359064,rs80359075,rs80359076,rs80359099,rs80359140,rs80359144,rs80359159,rs80359200,rs80359212,rs80359230,rs80359272,rs80359277,rs80359281,rs80359282,rs80359283,rs80359308,rs80359312,rs80359315,rs80359316,rs80359328,rs80359340,rs80359351,rs80359356,rs80359371,rs80359383,rs80359388,rs80359391,rs80359395,rs80359401,rs80359405,rs80359438,rs80359444,rs80359460,rs80359461,rs80359462,rs80359470,rs80359479,rs80359480,rs80359484,rs80359487,rs80359496,rs80359503,rs80359505,rs80359507,rs80359511,rs80359512,rs80359520,rs80359550,rs80359596,rs80359598,rs80359601,rs80359603,rs80359604,rs80359623,rs80359629,rs80359636,rs80359646,rs80359659,rs80359679,rs80359705,rs80359718,rs80359730,rs80359743,rs80359753,rs80359763,rs80359775,rs863225466,rs864622389,rs864622649,rs876659258,rs879255306 | 0.00001 |
| Homologous DNA Pairing and Strand Exchange | DNA Repair | *ATM,BRCA1,BRCA2,EXO1,PALB2,RAD51B* | rs11571658,rs11571833,rs11624333,rs118203998,rs118203999,rs121434219,rs1800057,rs1801516,rs180177083,rs180177099,rs180177100,rs180177110,rs180177132,rs180177133,rs180177142,rs180177143,rs201523522,rs201963507,rs202206540,rs2588809,rs273899695,rs273901751,rs28897672,rs28897696,rs28897743,rs28897756,rs28897758,rs28904921,rs371638537,rs376603775,rs397507239,rs397507320,rs397507331,rs397507333,rs397507404,rs397507582,rs397507686,rs397507713,rs397507762,rs397507868,rs397507943,rs397508034,rs397509267,rs397515635,rs398122546,rs398122602,rs398122749,rs398122772,rs41293455,rs41293459,rs41293461,rs41293463,rs41293465,rs41293477,rs41293497,rs41293511,rs41293513,rs431825343,rs45444999,rs45476495,rs45553935,rs45580035,rs515726117,rs532480170,rs55770810,rs564652222,rs587776408,rs587776410,rs587776411,rs587776416,rs587776527,rs587779815,rs587779818,rs587779836,rs587779844,rs587779846,rs587779865,rs587779869,rs587779872,rs587780210,rs587781347,rs587781598,rs587781658,rs587781730,rs587781776,rs587781905,rs587782005,rs587782081,rs587782173,rs587782198,rs587782240,rs587782292,rs587782652,rs587782680,rs587782719,rs62625303,rs62625308,rs72755295,rs730881304,rs730881348,rs730881359,rs730881391,rs730881394,rs730882166,rs747070579,rs747727055,rs748634900,rs749036865,rs75096777,rs758588019,rs762083530,rs763470424,rs764925691,rs769523686,rs770641163,rs772821016,rs776294034,rs778269655,rs779278389,rs780905851,rs786201675,rs786201689,rs786201693,rs786201957,rs786202239,rs786203008,rs786203054,rs786203272,rs786203370,rs786203488,rs786203606,rs786203714,rs786204273,rs786204433,rs786204751,rs80356885,rs80356898,rs80356929,rs80356978,rs80356991,rs80357018,rs80357243,rs80357426,rs80357508,rs80357516,rs80357522,rs80357542,rs80357572,rs80357573,rs80357687,rs80357723,rs80357729,rs80357760,rs80357813,rs80357853,rs80357868,rs80357888,rs80357906,rs80357939,rs80357960,rs80357968,rs80357970,rs80357971,rs80358094,rs80358504,rs80358523,rs80358533,rs80358556,rs80358579,rs80358620,rs80358710,rs80358721,rs80358810,rs80358824,rs80358843,rs80358972,rs80358981,rs80359003,rs80359022,rs80359031,rs80359064,rs80359075,rs80359076,rs80359099,rs80359140,rs80359144,rs80359159,rs80359200,rs80359212,rs80359230,rs80359272,rs80359277,rs80359281,rs80359282,rs80359283,rs80359308,rs80359312,rs80359315,rs80359316,rs80359328,rs80359340,rs80359351,rs80359356,rs80359371,rs80359383,rs80359388,rs80359391,rs80359395,rs80359401,rs80359405,rs80359438,rs80359444,rs80359460,rs80359461,rs80359462,rs80359470,rs80359479,rs80359480,rs80359484,rs80359487,rs80359496,rs80359503,rs80359505,rs80359507,rs80359511,rs80359512,rs80359520,rs80359550,rs80359596,rs80359598,rs80359601,rs80359603,rs80359604,rs80359623,rs80359629,rs80359636,rs80359646,rs80359659,rs80359679,rs80359705,rs80359718,rs80359730,rs80359743,rs80359753,rs80359763,rs80359775,rs863225466,rs864622389,rs864622649,rs876659258,rs879255306 | 0.000037 |
| Intrinsic Pathway for Apoptosis | Programmed Cell Death | *AKT1,BCL2L11,CASP8,CDKN2A,STAT3,TP53* | rs111736126,rs113050728,rs12613243,rs12951971,rs17041868,rs17041869,rs17881320,rs17881940,rs2494734,rs2498794,rs2811710,rs35850753,rs3761706,rs3769823,rs3789066,rs3789067,rs66812455,rs71801447,rs72948328,rs73954925,rs73954926,rs73954941,rs73954943,rs75543804,rs78378222,rs80130556 | 0.000155 |
| HDR through Homologous Recombination (HRR) or Single Strand Annealing (SSA) | DNA Repair | *ABRAXAS1,ATM,BRCA1,BRCA2,EXO1,HIST1H2BF,PALB2,RAD51B,TIPIN* | rs10008742,rs10012248,rs10015939,rs10016216,rs10023702,rs10025004,rs10027007,rs10032585,rs10033600,rs10213169,rs11099601,rs11571658,rs11571833,rs11624333,rs11727406,rs11728733,rs118203998,rs118203999,rs11930653,rs11935455,rs11940256,rs11947695,rs121434219,rs12649417,rs13112255,rs13121249,rs13128266,rs13128503,rs13129676,rs13139169,rs13142756,rs143767430,rs1565909,rs17352824,rs1800057,rs1801516,rs180177083,rs180177099,rs180177100,rs180177110,rs180177132,rs180177133,rs180177142,rs180177143,rs1964010,rs1980271,rs201523522,rs201963507,rs202206540,rs2132096,rs2132097,rs2172809,rs2177034,rs2588809,rs273899695,rs273901751,rs28488925,rs28603076,rs28653564,rs28783127,rs28785823,rs28869016,rs28869596,rs28875243,rs28895925,rs28897672,rs28897696,rs28897743,rs28897756,rs28897758,rs28904921,rs34136446,rs34803094,rs34961555,rs35000866,rs35452111,rs35608460,rs36014997,rs371638537,rs376603775,rs397507239,rs397507320,rs397507331,rs397507333,rs397507404,rs397507582,rs397507686,rs397507713,rs397507762,rs397507868,rs397507943,rs397508034,rs397509267,rs397515635,rs398122546,rs398122602,rs398122749,rs398122772,rs41293455,rs41293459,rs41293461,rs41293463,rs41293465,rs41293477,rs41293497,rs41293511,rs41293513,rs431825343,rs45444999,rs45476495,rs45553935,rs45580035,rs4693090,rs515726117,rs526064,rs532480170,rs538078,rs543318,rs555348,rs55770810,rs562723,rs564652222,rs568978,rs587776408,rs587776410,rs587776411,rs587776416,rs587776527,rs587779815,rs587779818,rs587779836,rs587779844,rs587779846,rs587779865,rs587779869,rs587779872,rs587780210,rs587781347,rs587781598,rs587781658,rs587781730,rs587781776,rs587781905,rs587782005,rs587782081,rs587782173,rs587782198,rs587782240,rs587782292,rs587782652,rs587782680,rs587782719,rs59644543,rs62625303,rs62625308,rs6535478,rs6834006,rs6838225,rs6844460,rs72755295,rs730881304,rs730881348,rs730881359,rs730881391,rs730881394,rs730882166,rs747070579,rs747727055,rs748634900,rs749036865,rs75096777,rs758588019,rs762083530,rs763470424,rs764925691,rs7663427,rs7666601,rs7671387,rs7691492,rs7694435,rs769523686,rs770641163,rs772821016,rs776294034,rs778269655,rs779278389,rs780905851,rs786201675,rs786201689,rs786201693,rs786201957,rs786202239,rs786203008,rs786203054,rs786203272,rs786203370,rs786203488,rs786203606,rs786203714,rs786204273,rs786204433,rs786204751,rs80356885,rs80356898,rs80356929,rs80356978,rs80356991,rs80357018,rs80357243,rs80357426,rs80357508,rs80357516,rs80357522,rs80357542,rs80357572,rs80357573,rs80357687,rs80357723,rs80357729,rs80357760,rs80357813,rs80357853,rs80357868,rs80357888,rs80357906,rs80357939,rs80357960,rs80357968,rs80357970,rs80357971,rs80358094,rs80358504,rs80358523,rs80358533,rs80358556,rs80358579,rs80358620,rs80358710,rs80358721,rs80358810,rs80358824,rs80358843,rs80358972,rs80358981,rs80359003,rs80359022,rs80359031,rs80359064,rs80359075,rs80359076,rs80359099,rs80359140,rs80359144,rs80359159,rs80359200,rs80359212,rs80359230,rs80359272,rs80359277,rs80359281,rs80359282,rs80359283,rs80359308,rs80359312,rs80359315,rs80359316,rs80359328,rs80359340,rs80359351,rs80359356,rs80359371,rs80359383,rs80359388,rs80359391,rs80359395,rs80359401,rs80359405,rs80359438,rs80359444,rs80359460,rs80359461,rs80359462,rs80359470,rs80359479,rs80359480,rs80359484,rs80359487,rs80359496,rs80359503,rs80359505,rs80359507,rs80359511,rs80359512,rs80359520,rs80359550,rs80359596,rs80359598,rs80359601,rs80359603,rs80359604,rs80359623,rs80359629,rs80359636,rs80359646,rs80359659,rs80359679,rs80359705,rs80359718,rs80359730,rs80359743,rs80359753,rs80359763,rs80359775,rs8042593,rs813298,rs863225466,rs864622389,rs864622649,rs876659258,rs879255306,rs9284657,rs9683689,rs971539,rs9997925 | 0.000172 |
| Regulation of TP53 Degradation | Gene expression (Transcription) | *AKT1,ATM,CDKN2A,CHEK2,TP53* | rs121434219,rs137853007,rs137853011,rs141568342,rs142763740,rs17879961,rs1800057,rs1801516,rs200432447,rs200928781,rs201963507,rs202206540,rs2494734,rs2498794,rs2811710,rs28904921,rs28909982,rs35850753,rs371638537,rs376603775,rs532480170,rs536907995,rs555607708,rs564652222,rs587779815,rs587779818,rs587779836,rs587779844,rs587779846,rs587779865,rs587779869,rs587779872,rs587780174,rs587781269,rs587781347,rs587781598,rs587781658,rs587781730,rs587781776,rs587781905,rs587782070,rs587782198,rs587782245,rs587782292,rs587782471,rs587782652,rs587782719,rs5997390,rs72552322,rs730881304,rs730881348,rs730881359,rs730881391,rs730881394,rs730881699,rs730881700,rs730881701,rs747727055,rs748634900,rs749036865,rs758588019,rs760502479,rs761494650,rs762083530,rs763470424,rs769523686,rs770641163,rs772821016,rs774175654,rs778269655,rs780905851,rs78378222,rs786201675,rs786201689,rs786201693,rs786201957,rs786202497,rs786203008,rs786203053,rs786203054,rs786203272,rs786203370,rs786203606,rs786204433,rs786204751,rs796389290,rs863225466,rs864622389,rs864622649 | 0.000192 |
| Regulation of TP53 Expression and Degradation | Gene expression (Transcription) | *AKT1,ATM,CDKN2A,CHEK2,TP53* | rs121434219,rs137853007,rs137853011,rs141568342,rs142763740,rs17879961,rs1800057,rs1801516,rs200432447,rs200928781,rs201963507,rs202206540,rs2494734,rs2498794,rs2811710,rs28904921,rs28909982,rs35850753,rs371638537,rs376603775,rs532480170,rs536907995,rs555607708,rs564652222,rs587779815,rs587779818,rs587779836,rs587779844,rs587779846,rs587779865,rs587779869,rs587779872,rs587780174,rs587781269,rs587781347,rs587781598,rs587781658,rs587781730,rs587781776,rs587781905,rs587782070,rs587782198,rs587782245,rs587782292,rs587782471,rs587782652,rs587782719,rs5997390,rs72552322,rs730881304,rs730881348,rs730881359,rs730881391,rs730881394,rs730881699,rs730881700,rs730881701,rs747727055,rs748634900,rs749036865,rs758588019,rs760502479,rs761494650,rs762083530,rs763470424,rs769523686,rs770641163,rs772821016,rs774175654,rs778269655,rs780905851,rs78378222,rs786201675,rs786201689,rs786201693,rs786201957,rs786202497,rs786203008,rs786203053,rs786203054,rs786203272,rs786203370,rs786203606,rs786204433,rs786204751,rs796389290,rs863225466,rs864622389,rs864622649 | 0.00022 |
| Homology Directed Repair | DNA Repair | *ABRAXAS1,ATM,BRCA1,BRCA2,EXO1,HIST1H2BF,PALB2,RAD51B,TIPIN* | rs10008742,rs10012248,rs10015939,rs10016216,rs10023702,rs10025004,rs10027007,rs10032585,rs10033600,rs10213169,rs11099601,rs11571658,rs11571833,rs11624333,rs11727406,rs11728733,rs118203998,rs118203999,rs11930653,rs11935455,rs11940256,rs11947695,rs121434219,rs12649417,rs13112255,rs13121249,rs13128266,rs13128503,rs13129676,rs13139169,rs13142756,rs143767430,rs1565909,rs17352824,rs1800057,rs1801516,rs180177083,rs180177099,rs180177100,rs180177110,rs180177132,rs180177133,rs180177142,rs180177143,rs1964010,rs1980271,rs201523522,rs201963507,rs202206540,rs2132096,rs2132097,rs2172809,rs2177034,rs2588809,rs273899695,rs273901751,rs28488925,rs28603076,rs28653564,rs28783127,rs28785823,rs28869016,rs28869596,rs28875243,rs28895925,rs28897672,rs28897696,rs28897743,rs28897756,rs28897758,rs28904921,rs34136446,rs34803094,rs34961555,rs35000866,rs35452111,rs35608460,rs36014997,rs371638537,rs376603775,rs397507239,rs397507320,rs397507331,rs397507333,rs397507404,rs397507582,rs397507686,rs397507713,rs397507762,rs397507868,rs397507943,rs397508034,rs397509267,rs397515635,rs398122546,rs398122602,rs398122749,rs398122772,rs41293455,rs41293459,rs41293461,rs41293463,rs41293465,rs41293477,rs41293497,rs41293511,rs41293513,rs431825343,rs45444999,rs45476495,rs45553935,rs45580035,rs4693090,rs515726117,rs526064,rs532480170,rs538078,rs543318,rs555348,rs55770810,rs562723,rs564652222,rs568978,rs587776408,rs587776410,rs587776411,rs587776416,rs587776527,rs587779815,rs587779818,rs587779836,rs587779844,rs587779846,rs587779865,rs587779869,rs587779872,rs587780210,rs587781347,rs587781598,rs587781658,rs587781730,rs587781776,rs587781905,rs587782005,rs587782081,rs587782173,rs587782198,rs587782240,rs587782292,rs587782652,rs587782680,rs587782719,rs59644543,rs62625303,rs62625308,rs6535478,rs6834006,rs6838225,rs6844460,rs72755295,rs730881304,rs730881348,rs730881359,rs730881391,rs730881394,rs730882166,rs747070579,rs747727055,rs748634900,rs749036865,rs75096777,rs758588019,rs762083530,rs763470424,rs764925691,rs7663427,rs7666601,rs7671387,rs7691492,rs7694435,rs769523686,rs770641163,rs772821016,rs776294034,rs778269655,rs779278389,rs780905851,rs786201675,rs786201689,rs786201693,rs786201957,rs786202239,rs786203008,rs786203054,rs786203272,rs786203370,rs786203488,rs786203606,rs786203714,rs786204273,rs786204433,rs786204751,rs80356885,rs80356898,rs80356929,rs80356978,rs80356991,rs80357018,rs80357243,rs80357426,rs80357508,rs80357516,rs80357522,rs80357542,rs80357572,rs80357573,rs80357687,rs80357723,rs80357729,rs80357760,rs80357813,rs80357853,rs80357868,rs80357888,rs80357906,rs80357939,rs80357960,rs80357968,rs80357970,rs80357971,rs80358094,rs80358504,rs80358523,rs80358533,rs80358556,rs80358579,rs80358620,rs80358710,rs80358721,rs80358810,rs80358824,rs80358843,rs80358972,rs80358981,rs80359003,rs80359022,rs80359031,rs80359064,rs80359075,rs80359076,rs80359099,rs80359140,rs80359144,rs80359159,rs80359200,rs80359212,rs80359230,rs80359272,rs80359277,rs80359281,rs80359282,rs80359283,rs80359308,rs80359312,rs80359315,rs80359316,rs80359328,rs80359340,rs80359351,rs80359356,rs80359371,rs80359383,rs80359388,rs80359391,rs80359395,rs80359401,rs80359405,rs80359438,rs80359444,rs80359460,rs80359461,rs80359462,rs80359470,rs80359479,rs80359480,rs80359484,rs80359487,rs80359496,rs80359503,rs80359505,rs80359507,rs80359511,rs80359512,rs80359520,rs80359550,rs80359596,rs80359598,rs80359601,rs80359603,rs80359604,rs80359623,rs80359629,rs80359636,rs80359646,rs80359659,rs80359679,rs80359705,rs80359718,rs80359730,rs80359743,rs80359753,rs80359763,rs80359775,rs8042593,rs813298,rs863225466,rs864622389,rs864622649,rs876659258,rs879255306,rs9284657,rs9683689,rs971539,rs9997925 | 0.000241 |
| Transcriptional regulation by the AP-2 (TFAP2) family of transcription factors | Gene expression (Transcription) | *APOE,CGA,EGFR,ESR1,KCTD1* | rs1111207,rs13244925,rs57589542,rs6904031,rs73754909,rs7412,rs745338901,rs77846138,rs851984,rs910416 | 0.00025 |
| Presynaptic phase of homologous DNA pairing and strand exchange | DNA Repair | *ATM,BRCA1,BRCA2,EXO1,RAD51B* | rs11571658,rs11571833,rs11624333,rs121434219,rs1800057,rs1801516,rs201523522,rs201963507,rs202206540,rs2588809,rs273899695,rs273901751,rs28897672,rs28897696,rs28897743,rs28897756,rs28897758,rs28904921,rs371638537,rs376603775,rs397507239,rs397507320,rs397507331,rs397507333,rs397507404,rs397507582,rs397507686,rs397507713,rs397507762,rs397507868,rs397507943,rs397508034,rs397509267,rs397515635,rs398122546,rs398122602,rs398122749,rs398122772,rs41293455,rs41293459,rs41293461,rs41293463,rs41293465,rs41293477,rs41293497,rs41293511,rs41293513,rs431825343,rs45444999,rs45553935,rs45580035,rs532480170,rs55770810,rs564652222,rs587779815,rs587779818,rs587779836,rs587779844,rs587779846,rs587779865,rs587779869,rs587779872,rs587781347,rs587781598,rs587781658,rs587781730,rs587781776,rs587781905,rs587782173,rs587782198,rs587782240,rs587782292,rs587782652,rs587782719,rs62625303,rs62625308,rs72755295,rs730881304,rs730881348,rs730881359,rs730881391,rs730881394,rs730882166,rs747070579,rs747727055,rs748634900,rs749036865,rs75096777,rs758588019,rs762083530,rs763470424,rs769523686,rs770641163,rs772821016,rs778269655,rs780905851,rs786201675,rs786201689,rs786201693,rs786201957,rs786203008,rs786203054,rs786203272,rs786203370,rs786203606,rs786204273,rs786204433,rs786204751,rs80356885,rs80356898,rs80356929,rs80356978,rs80356991,rs80357018,rs80357243,rs80357426,rs80357508,rs80357516,rs80357522,rs80357542,rs80357572,rs80357573,rs80357687,rs80357723,rs80357729,rs80357760,rs80357813,rs80357853,rs80357868,rs80357888,rs80357906,rs80357939,rs80357960,rs80357968,rs80357970,rs80357971,rs80358094,rs80358504,rs80358523,rs80358533,rs80358556,rs80358579,rs80358620,rs80358710,rs80358721,rs80358810,rs80358824,rs80358843,rs80358972,rs80358981,rs80359003,rs80359022,rs80359031,rs80359064,rs80359075,rs80359076,rs80359099,rs80359140,rs80359144,rs80359159,rs80359200,rs80359212,rs80359230,rs80359272,rs80359277,rs80359281,rs80359282,rs80359283,rs80359308,rs80359312,rs80359315,rs80359316,rs80359328,rs80359340,rs80359351,rs80359356,rs80359371,rs80359383,rs80359388,rs80359391,rs80359395,rs80359401,rs80359405,rs80359438,rs80359444,rs80359460,rs80359461,rs80359462,rs80359470,rs80359479,rs80359480,rs80359484,rs80359487,rs80359496,rs80359503,rs80359505,rs80359507,rs80359511,rs80359512,rs80359520,rs80359550,rs80359596,rs80359598,rs80359601,rs80359603,rs80359604,rs80359623,rs80359629,rs80359636,rs80359646,rs80359659,rs80359679,rs80359705,rs80359718,rs80359730,rs80359743,rs80359753,rs80359763,rs80359775,rs863225466,rs864622389,rs864622649,rs876659258,rs879255306 | 0.000283 |
| DNA Repair | DNA Repair | *ABRAXAS1,ATM,BRCA1,BRCA2,CHEK2,ELL,EXO1,FTO,HIST1H2BF,PALB2,RAD51B,TIPIN,TP53,XRCC6* | rs10008742,rs10012248,rs10015939,rs10016216,rs10023702,rs10025004,rs10027007,rs10032585,rs10033600,rs10164323,rs10213169,rs11099601,rs11571658,rs11571833,rs11624333,rs11727406,rs11728733,rs118203998,rs118203999,rs11930653,rs11935455,rs11940256,rs11947695,rs121434219,rs12649417,rs13112255,rs13121249,rs13128266,rs13128503,rs13129676,rs13139169,rs13142756,rs137853007,rs137853011,rs141568342,rs142763740,rs143767430,rs1565909,rs17352824,rs17879961,rs1800057,rs1801516,rs180177083,rs180177099,rs180177100,rs180177110,rs180177132,rs180177133,rs180177142,rs180177143,rs1964010,rs1980271,rs200432447,rs200928781,rs201523522,rs201963507,rs202206540,rs2132096,rs2132097,rs2172809,rs2177034,rs2588809,rs273899695,rs273901751,rs28488925,rs28603076,rs28653564,rs28783127,rs28785823,rs28869016,rs28869596,rs28875243,rs28895925,rs28897672,rs28897696,rs28897743,rs28897756,rs28897758,rs28904921,rs28909982,rs34136446,rs34803094,rs34961555,rs35000866,rs35452111,rs35608460,rs35850753,rs36014997,rs371638537,rs376603775,rs397507239,rs397507320,rs397507331,rs397507333,rs397507404,rs397507582,rs397507686,rs397507713,rs397507762,rs397507868,rs397507943,rs397508034,rs397509267,rs397515635,rs398122546,rs398122602,rs398122749,rs398122772,rs41293455,rs41293459,rs41293461,rs41293463,rs41293465,rs41293477,rs41293497,rs41293511,rs41293513,rs431825343,rs45444999,rs45476495,rs45553935,rs45580035,rs4693090,rs515726117,rs526064,rs532480170,rs536907995,rs538078,rs543318,rs555348,rs555607708,rs55770810,rs55872725,rs562723,rs564652222,rs568978,rs587776408,rs587776410,rs587776411,rs587776416,rs587776527,rs587779815,rs587779818,rs587779836,rs587779844,rs587779846,rs587779865,rs587779869,rs587779872,rs587780174,rs587780210,rs587781269,rs587781347,rs587781598,rs587781658,rs587781730,rs587781776,rs587781905,rs587782005,rs587782070,rs587782081,rs587782173,rs587782198,rs587782240,rs587782245,rs587782292,rs587782471,rs587782652,rs587782680,rs587782719,rs59644543,rs5997390,rs62625303,rs62625308,rs6499648,rs6535478,rs6834006,rs6838225,rs6844460,rs7184573,rs72552322,rs72755295,rs730881304,rs730881348,rs730881359,rs730881391,rs730881394,rs730881699,rs730881700,rs730881701,rs730882166,rs73161324,rs747070579,rs747727055,rs748634900,rs749036865,rs75096777,rs758588019,rs760502479,rs761494650,rs762083530,rs763470424,rs764925691,rs7663427,rs7666601,rs7671387,rs7691492,rs7694435,rs769523686,rs770641163,rs772821016,rs774175654,rs776294034,rs778269655,rs779278389,rs780905851,rs78378222,rs786201675,rs786201689,rs786201693,rs786201957,rs786202239,rs786202497,rs786203008,rs786203053,rs786203054,rs786203272,rs786203370,rs786203488,rs786203606,rs786203714,rs786204273,rs786204433,rs786204751,rs796389290,rs80356885,rs80356898,rs80356929,rs80356978,rs80356991,rs80357018,rs80357243,rs80357426,rs80357508,rs80357516,rs80357522,rs80357542,rs80357572,rs80357573,rs80357687,rs80357723,rs80357729,rs80357760,rs80357813,rs80357853,rs80357868,rs80357888,rs80357906,rs80357939,rs80357960,rs80357968,rs80357970,rs80357971,rs80358094,rs80358504,rs80358523,rs80358533,rs80358556,rs80358579,rs80358620,rs80358710,rs80358721,rs80358810,rs80358824,rs80358843,rs80358972,rs80358981,rs80359003,rs80359022,rs80359031,rs80359064,rs80359075,rs80359076,rs80359099,rs80359140,rs80359144,rs80359159,rs80359200,rs80359212,rs80359230,rs80359272,rs80359277,rs80359281,rs80359282,rs80359283,rs80359308,rs80359312,rs80359315,rs80359316,rs80359328,rs80359340,rs80359351,rs80359356,rs80359371,rs80359383,rs80359388,rs80359391,rs80359395,rs80359401,rs80359405,rs80359438,rs80359444,rs80359460,rs80359461,rs80359462,rs80359470,rs80359479,rs80359480,rs80359484,rs80359487,rs80359496,rs80359503,rs80359505,rs80359507,rs80359511,rs80359512,rs80359520,rs80359550,rs80359596,rs80359598,rs80359601,rs80359603,rs80359604,rs80359623,rs80359629,rs80359636,rs80359646,rs80359659,rs80359679,rs80359705,rs80359718,rs80359730,rs80359743,rs80359753,rs80359763,rs80359775,rs8042593,rs813298,rs863225466,rs864622389,rs864622649,rs876659258,rs879255306,rs9284657,rs9683689,rs971539,rs9997925 | 0.000492 |
| HDR through Homologous Recombination (HRR) | DNA Repair | *ATM,BRCA1,BRCA2,EXO1,PALB2,RAD51B* | rs11571658,rs11571833,rs11624333,rs118203998,rs118203999,rs121434219,rs1800057,rs1801516,rs180177083,rs180177099,rs180177100,rs180177110,rs180177132,rs180177133,rs180177142,rs180177143,rs201523522,rs201963507,rs202206540,rs2588809,rs273899695,rs273901751,rs28897672,rs28897696,rs28897743,rs28897756,rs28897758,rs28904921,rs371638537,rs376603775,rs397507239,rs397507320,rs397507331,rs397507333,rs397507404,rs397507582,rs397507686,rs397507713,rs397507762,rs397507868,rs397507943,rs397508034,rs397509267,rs397515635,rs398122546,rs398122602,rs398122749,rs398122772,rs41293455,rs41293459,rs41293461,rs41293463,rs41293465,rs41293477,rs41293497,rs41293511,rs41293513,rs431825343,rs45444999,rs45476495,rs45553935,rs45580035,rs515726117,rs532480170,rs55770810,rs564652222,rs587776408,rs587776410,rs587776411,rs587776416,rs587776527,rs587779815,rs587779818,rs587779836,rs587779844,rs587779846,rs587779865,rs587779869,rs587779872,rs587780210,rs587781347,rs587781598,rs587781658,rs587781730,rs587781776,rs587781905,rs587782005,rs587782081,rs587782173,rs587782198,rs587782240,rs587782292,rs587782652,rs587782680,rs587782719,rs62625303,rs62625308,rs72755295,rs730881304,rs730881348,rs730881359,rs730881391,rs730881394,rs730882166,rs747070579,rs747727055,rs748634900,rs749036865,rs75096777,rs758588019,rs762083530,rs763470424,rs764925691,rs769523686,rs770641163,rs772821016,rs776294034,rs778269655,rs779278389,rs780905851,rs786201675,rs786201689,rs786201693,rs786201957,rs786202239,rs786203008,rs786203054,rs786203272,rs786203370,rs786203488,rs786203606,rs786203714,rs786204273,rs786204433,rs786204751,rs80356885,rs80356898,rs80356929,rs80356978,rs80356991,rs80357018,rs80357243,rs80357426,rs80357508,rs80357516,rs80357522,rs80357542,rs80357572,rs80357573,rs80357687,rs80357723,rs80357729,rs80357760,rs80357813,rs80357853,rs80357868,rs80357888,rs80357906,rs80357939,rs80357960,rs80357968,rs80357970,rs80357971,rs80358094,rs80358504,rs80358523,rs80358533,rs80358556,rs80358579,rs80358620,rs80358710,rs80358721,rs80358810,rs80358824,rs80358843,rs80358972,rs80358981,rs80359003,rs80359022,rs80359031,rs80359064,rs80359075,rs80359076,rs80359099,rs80359140,rs80359144,rs80359159,rs80359200,rs80359212,rs80359230,rs80359272,rs80359277,rs80359281,rs80359282,rs80359283,rs80359308,rs80359312,rs80359315,rs80359316,rs80359328,rs80359340,rs80359351,rs80359356,rs80359371,rs80359383,rs80359388,rs80359391,rs80359395,rs80359401,rs80359405,rs80359438,rs80359444,rs80359460,rs80359461,rs80359462,rs80359470,rs80359479,rs80359480,rs80359484,rs80359487,rs80359496,rs80359503,rs80359505,rs80359507,rs80359511,rs80359512,rs80359520,rs80359550,rs80359596,rs80359598,rs80359601,rs80359603,rs80359604,rs80359623,rs80359629,rs80359636,rs80359646,rs80359659,rs80359679,rs80359705,rs80359718,rs80359730,rs80359743,rs80359753,rs80359763,rs80359775,rs863225466,rs864622389,rs864622649,rs876659258,rs879255306 | 0.000509 |
| Diseases of signal transduction by growth factor receptors and second messengers | Disease | *AKT1,BCL2L11,CUX1,EGFR,ERBB4,ESR1,FGFR2,KDR,KREMEN1,MAP2K1,PIK3R3,SMAD3,STAT3,TCF7L2,TGFBR2* | rs1085240,rs10885405,rs111572611,rs111736126,rs112293876,rs112919607,rs113050728,rs11972884,rs1219648,rs12613243,rs12951971,rs132289,rs13229095,rs13244925,rs143524711,rs1588663,rs1612419,rs1613296,rs17041868,rs17041869,rs1707303,rs1707317,rs1707337,rs1707338,rs1707339,rs1768800,rs1768801,rs1768802,rs1768807,rs1768815,rs1768817,rs1768818,rs17838698,rs17881320,rs17881940,rs190740846,rs2297883,rs2458400,rs2494734,rs2498794,rs2981575,rs2981579,rs2981584,rs34231037,rs34234237,rs35378730,rs35808728,rs35874463,rs3761706,rs3789066,rs3789067,rs397897588,rs45631563,rs45631580,rs55985922,rs57589542,rs5773899,rs59197560,rs66812455,rs67716739,rs6904031,rs6961094,rs6979850,rs71559437,rs71801447,rs72948328,rs73954925,rs73954926,rs73954941,rs73954943,rs745338901,rs75543804,rs7796917,rs785462,rs785463,rs785465,rs785466,rs785467,rs785468,rs785469,rs785470,rs785483,rs785484,rs785486,rs785490,rs785493,rs785496,rs785497,rs785498,rs785499,rs785500,rs785501,rs785504,rs785506,rs785507,rs785508,rs785509,rs785510,rs785512,rs785513,rs785516,rs785517,rs785518,rs785519,rs796773,rs80130556,rs809774,rs814168,rs851984,rs910416,rs9429186 | 0.000541 |
| G2/M DNA damage checkpoint | Cell Cycle | *ABRAXAS1,ATM,BRCA1,CHEK2,EXO1,HIST1H2BF,TP53* | rs10008742,rs10012248,rs10015939,rs10016216,rs10023702,rs10025004,rs10027007,rs10032585,rs10033600,rs10213169,rs11099601,rs11727406,rs11728733,rs11930653,rs11935455,rs11940256,rs11947695,rs121434219,rs12649417,rs13112255,rs13121249,rs13128266,rs13128503,rs13129676,rs13139169,rs13142756,rs137853007,rs137853011,rs141568342,rs142763740,rs143767430,rs1565909,rs17352824,rs17879961,rs1800057,rs1801516,rs1964010,rs1980271,rs200432447,rs200928781,rs201963507,rs202206540,rs2132096,rs2132097,rs2172809,rs2177034,rs273899695,rs273901751,rs28488925,rs28603076,rs28653564,rs28783127,rs28785823,rs28869016,rs28869596,rs28875243,rs28895925,rs28897672,rs28897696,rs28904921,rs28909982,rs34136446,rs34803094,rs34961555,rs35000866,rs35452111,rs35608460,rs35850753,rs36014997,rs371638537,rs376603775,rs397507239,rs397509267,rs41293455,rs41293459,rs41293461,rs41293463,rs41293465,rs45444999,rs45553935,rs4693090,rs526064,rs532480170,rs536907995,rs538078,rs543318,rs555348,rs555607708,rs55770810,rs562723,rs564652222,rs568978,rs587779815,rs587779818,rs587779836,rs587779844,rs587779846,rs587779865,rs587779869,rs587779872,rs587780174,rs587781269,rs587781347,rs587781598,rs587781658,rs587781730,rs587781776,rs587781905,rs587782070,rs587782173,rs587782198,rs587782245,rs587782292,rs587782471,rs587782652,rs587782719,rs59644543,rs5997390,rs62625303,rs62625308,rs6535478,rs6834006,rs6838225,rs6844460,rs72552322,rs72755295,rs730881304,rs730881348,rs730881359,rs730881391,rs730881394,rs730881699,rs730881700,rs730881701,rs730882166,rs747727055,rs748634900,rs749036865,rs758588019,rs760502479,rs761494650,rs762083530,rs763470424,rs7663427,rs7666601,rs7671387,rs7691492,rs7694435,rs769523686,rs770641163,rs772821016,rs774175654,rs778269655,rs780905851,rs78378222,rs786201675,rs786201689,rs786201693,rs786201957,rs786202497,rs786203008,rs786203053,rs786203054,rs786203272,rs786203370,rs786203606,rs786204433,rs786204751,rs796389290,rs80356885,rs80356898,rs80356929,rs80356978,rs80356991,rs80357018,rs80357243,rs80357426,rs80357508,rs80357516,rs80357522,rs80357542,rs80357572,rs80357573,rs80357687,rs80357723,rs80357729,rs80357760,rs80357813,rs80357853,rs80357868,rs80357888,rs80357906,rs80357939,rs80357960,rs80357968,rs80357970,rs80357971,rs80358094,rs813298,rs863225466,rs864622389,rs864622649,rs9284657,rs9683689,rs971539,rs9997925 | 0.000577 |
| TP53 Regulates Transcription of Caspase Activators and Caspases | Gene expression (Transcription) | *ATM,PIDD1,TP53* | rs10902221,rs11246313,rs11246314,rs11246316,rs11246318,rs121434219,rs1800057,rs1801516,rs201963507,rs202206540,rs28904921,rs35850753,rs371638537,rs376603775,rs532480170,rs564652222,rs587779815,rs587779818,rs587779836,rs587779844,rs587779846,rs587779865,rs587779869,rs587779872,rs587781347,rs587781598,rs587781658,rs587781730,rs587781776,rs587781905,rs587782198,rs587782292,rs587782652,rs587782719,rs6597981,rs7104785,rs7117921,rs7121646,rs7122416,rs730881304,rs730881348,rs730881359,rs730881391,rs730881394,rs747727055,rs7479183,rs7484068,rs7484123,rs748634900,rs749036865,rs758588019,rs762083530,rs763470424,rs769523686,rs770641163,rs772821016,rs778269655,rs780905851,rs78378222,rs786201675,rs786201689,rs786201693,rs786201957,rs786203008,rs786203054,rs786203272,rs786203370,rs786203606,rs786204433,rs786204751,rs863225466,rs864622389,rs864622649 | 0.000676 |
| Regulation of TP53 Activity | Gene expression (Transcription) | *AKT1,ATM,BRCA1,CDKN2A,CHEK2,EXO1,GATAD2A,PIP4K2A,TP53* | rs1000237,rs10282,rs10402451,rs10405625,rs10408401,rs10410664,rs10414830,rs10419912,rs1047361,rs1054284,rs1063966,rs10764337,rs11459691,rs121434219,rs12975119,rs137853007,rs137853011,rs140702307,rs141568342,rs142763740,rs1469712,rs1469713,rs147477831,rs17879961,rs1800057,rs1801516,rs1808653,rs1858999,rs1865034,rs200432447,rs200928781,rs201963507,rs202206540,rs2082421,rs2099334,rs2288852,rs2315610,rs2494734,rs2498794,rs273899695,rs273901751,rs2811710,rs28897672,rs28897696,rs28904921,rs28909982,rs2916068,rs2916069,rs2916073,rs2916074,rs2916076,rs2965182,rs2965183,rs2965188,rs2965189,rs34027746,rs34349730,rs35524116,rs35850753,rs371638537,rs3752151,rs376603775,rs3794990,rs3794993,rs397507239,rs397509267,rs41293455,rs41293459,rs41293461,rs41293463,rs41293465,rs45444999,rs45553935,rs4808198,rs4808200,rs4808202,rs4808203,rs4808955,rs4808957,rs4808959,rs4808961,rs4808964,rs532480170,rs536907995,rs555607708,rs55770810,rs564652222,rs587779815,rs587779818,rs587779836,rs587779844,rs587779846,rs587779865,rs587779869,rs587779872,rs587780174,rs587781269,rs587781347,rs587781598,rs587781658,rs587781730,rs587781776,rs587781905,rs587782070,rs587782173,rs587782198,rs587782245,rs587782292,rs587782471,rs587782652,rs587782719,rs5997390,rs62625303,rs62625308,rs6511038,rs6626,rs6909,rs72552322,rs7256149,rs72755295,rs730881304,rs730881348,rs730881359,rs730881391,rs730881394,rs730881699,rs730881700,rs730881701,rs730882166,rs747050,rs747727055,rs748634900,rs749036865,rs751856,rs758588019,rs760502479,rs761494650,rs762083530,rs763470424,rs769523686,rs770641163,rs772821016,rs774175654,rs778269655,rs780905851,rs78378222,rs786201675,rs786201689,rs786201693,rs786201957,rs786202497,rs786203008,rs786203053,rs786203054,rs786203272,rs786203370,rs786203606,rs786204433,rs786204751,rs796389290,rs80356885,rs80356898,rs80356929,rs80356978,rs80356991,rs80357018,rs80357243,rs80357426,rs80357508,rs80357516,rs80357522,rs80357542,rs80357572,rs80357573,rs80357687,rs80357723,rs80357729,rs80357760,rs80357813,rs80357853,rs80357868,rs80357888,rs80357906,rs80357939,rs80357960,rs80357968,rs80357970,rs80357971,rs80358094,rs863225466,rs864622389,rs864622649,rs892021,rs892022,rs9917108 | 0.000714 |
| Cell Cycle | Cell Cycle | *ABRAXAS1,AKT1,ATM,BRCA1,BRCA2,CDC14A,CDKN2A,CHEK2,DSCC1,EXO1,HIST1H2BF,KNL1,MAD1L1,MAU2,MCM8,PCM1,PCNT,PRIM1,TERT,TP53,TUBG2,ZWILCH* | rs10008742,rs10012248,rs10015939,rs10016216,rs10023702,rs10025004,rs10027007,rs10032585,rs10033600,rs10069690,rs10213169,rs11071896,rs11099601,rs11571658,rs11571833,rs11727406,rs11728733,rs11930653,rs11935455,rs11940256,rs11947695,rs121434219,rs12649417,rs12699477,rs13112255,rs13121249,rs13128266,rs13128503,rs13129676,rs13139169,rs13142756,rs137853007,rs137853011,rs13964,rs141568342,rs142763740,rs143767430,rs15622,rs1565909,rs16991615,rs17352824,rs17879961,rs1800057,rs1801516,rs1964010,rs1980271,rs200432447,rs200928781,rs201523522,rs201963507,rs202206540,rs2074090,rs2074091,rs2132096,rs2132097,rs2172809,rs2177034,rs2242652,rs2277339,rs2494734,rs2498794,rs273899695,rs273901751,rs2811710,rs28488925,rs28603076,rs28653564,rs28783127,rs28785823,rs28869016,rs28869596,rs28875243,rs28895925,rs28897672,rs28897696,rs28897743,rs28897756,rs28897758,rs28904921,rs28909982,rs34136446,rs34803094,rs34961555,rs35000866,rs35452111,rs35608460,rs35850753,rs36014997,rs371638537,rs376603775,rs397507239,rs397507320,rs397507331,rs397507333,rs397507404,rs397507582,rs397507686,rs397507713,rs397507762,rs397507868,rs397507943,rs397508034,rs397509267,rs397515635,rs398122546,rs398122602,rs398122749,rs398122772,rs41293455,rs41293459,rs41293461,rs41293463,rs41293465,rs41293477,rs41293497,rs41293511,rs41293513,rs431825343,rs45444999,rs45553935,rs45580035,rs4693090,rs4818836,rs4924487,rs526064,rs527298592,rs527510716,rs532480170,rs536907995,rs538078,rs543318,rs555348,rs555607708,rs55770810,rs562723,rs564652222,rs568978,rs587779815,rs587779818,rs587779836,rs587779844,rs587779846,rs587779865,rs587779869,rs587779872,rs587780174,rs587781269,rs587781347,rs587781598,rs587781658,rs587781730,rs587781776,rs587781905,rs587782070,rs587782173,rs587782198,rs587782240,rs587782245,rs587782292,rs587782471,rs587782652,rs587782719,rs59644543,rs5997390,rs612683,rs62526620,rs62625303,rs62625308,rs6535478,rs6834006,rs6838225,rs6844460,rs72552322,rs72709458,rs72755295,rs730881304,rs730881348,rs730881359,rs730881391,rs730881394,rs730881699,rs730881700,rs730881701,rs730882166,rs747070579,rs747727055,rs748634900,rs749036865,rs75096777,rs758588019,rs760502479,rs761494650,rs762083530,rs763470424,rs7663427,rs7666601,rs7671387,rs7691492,rs769267,rs7694435,rs769523686,rs770641163,rs772821016,rs774175654,rs778269655,rs780905851,rs78378222,rs786201675,rs786201689,rs786201693,rs786201957,rs786202497,rs786203008,rs786203053,rs786203054,rs786203272,rs786203370,rs786203606,rs786204273,rs786204433,rs786204751,rs796389290,rs80356885,rs80356898,rs80356929,rs80356978,rs80356991,rs80357018,rs80357243,rs80357426,rs80357508,rs80357516,rs80357522,rs80357542,rs80357572,rs80357573,rs80357687,rs80357723,rs80357729,rs80357760,rs80357813,rs80357853,rs80357868,rs80357888,rs80357906,rs80357939,rs80357960,rs80357968,rs80357970,rs80357971,rs80358094,rs80358504,rs80358523,rs80358533,rs80358556,rs80358579,rs80358620,rs80358710,rs80358721,rs80358810,rs80358824,rs80358843,rs80358972,rs80358981,rs80359003,rs80359022,rs80359031,rs80359064,rs80359075,rs80359076,rs80359099,rs80359140,rs80359144,rs80359159,rs80359200,rs80359212,rs80359230,rs80359272,rs80359277,rs80359281,rs80359282,rs80359283,rs80359308,rs80359312,rs80359315,rs80359316,rs80359328,rs80359340,rs80359351,rs80359356,rs80359371,rs80359383,rs80359388,rs80359391,rs80359395,rs80359401,rs80359405,rs80359438,rs80359444,rs80359460,rs80359461,rs80359462,rs80359470,rs80359479,rs80359480,rs80359484,rs80359487,rs80359496,rs80359503,rs80359505,rs80359507,rs80359511,rs80359512,rs80359520,rs80359550,rs80359596,rs80359598,rs80359601,rs80359603,rs80359604,rs80359623,rs80359629,rs80359636,rs80359646,rs80359659,rs80359679,rs80359705,rs80359718,rs80359730,rs80359743,rs80359753,rs80359763,rs80359775,rs8111511,rs813298,rs863225466,rs864622389,rs864622649,rs876659258,rs879255306,rs9284657,rs9683689,rs971539,rs9897504,rs9997925 | 0.000795 |
| PI5P, PP2A and IER3 Regulate PI3K/AKT Signaling | Signal Transduction | *AKT1,EGFR,ERBB4,ESR1,FGFR2,PIK3R3,PIP4K2A* | rs10764337,rs1085240,rs112919607,rs1219648,rs13244925,rs143524711,rs1588663,rs1612419,rs1613296,rs1707303,rs1707317,rs1707337,rs1707338,rs1707339,rs1768800,rs1768801,rs1768802,rs1768807,rs1768815,rs1768817,rs1768818,rs190740846,rs2297883,rs2458400,rs2494734,rs2498794,rs2981575,rs2981579,rs2981584,rs34234237,rs35378730,rs35808728,rs397897588,rs45631563,rs45631580,rs55985922,rs57589542,rs5773899,rs59197560,rs67716739,rs6904031,rs745338901,rs785462,rs785463,rs785465,rs785466,rs785467,rs785468,rs785469,rs785470,rs785483,rs785484,rs785486,rs785490,rs785493,rs785496,rs785497,rs785498,rs785499,rs785500,rs785501,rs785504,rs785506,rs785507,rs785508,rs785509,rs785510,rs785512,rs785513,rs785516,rs785517,rs785518,rs785519,rs796773,rs809774,rs814168,rs851984,rs910416,rs9429186 | 0.000936 |
| Resolution of D-loop Structures through Synthesis-Dependent Strand Annealing (SDSA) | DNA Repair | *ATM,BRCA1,BRCA2,EXO1,PALB2,RAD51B* | rs11571658,rs11571833,rs11624333,rs118203998,rs118203999,rs121434219,rs1800057,rs1801516,rs180177083,rs180177099,rs180177100,rs180177110,rs180177132,rs180177133,rs180177142,rs180177143,rs201523522,rs201963507,rs202206540,rs2588809,rs273899695,rs273901751,rs28897672,rs28897696,rs28897743,rs28897756,rs28897758,rs28904921,rs371638537,rs376603775,rs397507239,rs397507320,rs397507331,rs397507333,rs397507404,rs397507582,rs397507686,rs397507713,rs397507762,rs397507868,rs397507943,rs397508034,rs397509267,rs397515635,rs398122546,rs398122602,rs398122749,rs398122772,rs41293455,rs41293459,rs41293461,rs41293463,rs41293465,rs41293477,rs41293497,rs41293511,rs41293513,rs431825343,rs45444999,rs45476495,rs45553935,rs45580035,rs515726117,rs532480170,rs55770810,rs564652222,rs587776408,rs587776410,rs587776411,rs587776416,rs587776527,rs587779815,rs587779818,rs587779836,rs587779844,rs587779846,rs587779865,rs587779869,rs587779872,rs587780210,rs587781347,rs587781598,rs587781658,rs587781730,rs587781776,rs587781905,rs587782005,rs587782081,rs587782173,rs587782198,rs587782240,rs587782292,rs587782652,rs587782680,rs587782719,rs62625303,rs62625308,rs72755295,rs730881304,rs730881348,rs730881359,rs730881391,rs730881394,rs730882166,rs747070579,rs747727055,rs748634900,rs749036865,rs75096777,rs758588019,rs762083530,rs763470424,rs764925691,rs769523686,rs770641163,rs772821016,rs776294034,rs778269655,rs779278389,rs780905851,rs786201675,rs786201689,rs786201693,rs786201957,rs786202239,rs786203008,rs786203054,rs786203272,rs786203370,rs786203488,rs786203606,rs786203714,rs786204273,rs786204433,rs786204751,rs80356885,rs80356898,rs80356929,rs80356978,rs80356991,rs80357018,rs80357243,rs80357426,rs80357508,rs80357516,rs80357522,rs80357542,rs80357572,rs80357573,rs80357687,rs80357723,rs80357729,rs80357760,rs80357813,rs80357853,rs80357868,rs80357888,rs80357906,rs80357939,rs80357960,rs80357968,rs80357970,rs80357971,rs80358094,rs80358504,rs80358523,rs80358533,rs80358556,rs80358579,rs80358620,rs80358710,rs80358721,rs80358810,rs80358824,rs80358843,rs80358972,rs80358981,rs80359003,rs80359022,rs80359031,rs80359064,rs80359075,rs80359076,rs80359099,rs80359140,rs80359144,rs80359159,rs80359200,rs80359212,rs80359230,rs80359272,rs80359277,rs80359281,rs80359282,rs80359283,rs80359308,rs80359312,rs80359315,rs80359316,rs80359328,rs80359340,rs80359351,rs80359356,rs80359371,rs80359383,rs80359388,rs80359391,rs80359395,rs80359401,rs80359405,rs80359438,rs80359444,rs80359460,rs80359461,rs80359462,rs80359470,rs80359479,rs80359480,rs80359484,rs80359487,rs80359496,rs80359503,rs80359505,rs80359507,rs80359511,rs80359512,rs80359520,rs80359550,rs80359596,rs80359598,rs80359601,rs80359603,rs80359604,rs80359623,rs80359629,rs80359636,rs80359646,rs80359659,rs80359679,rs80359705,rs80359718,rs80359730,rs80359743,rs80359753,rs80359763,rs80359775,rs863225466,rs864622389,rs864622649,rs876659258,rs879255306 | 0.000002 |
| DNA Double-Strand Break Repair | DNA Repair | *ABRAXAS1,ATM,BRCA1,BRCA2,CHEK2,EXO1,HIST1H2BF,PALB2,RAD51B,TIPIN,TP53,XRCC6* | rs10008742,rs10012248,rs10015939,rs10016216,rs10023702,rs10025004,rs10027007,rs10032585,rs10033600,rs10213169,rs11099601,rs11571658,rs11571833,rs11624333,rs11727406,rs11728733,rs118203998,rs118203999,rs11930653,rs11935455,rs11940256,rs11947695,rs121434219,rs12649417,rs13112255,rs13121249,rs13128266,rs13128503,rs13129676,rs13139169,rs13142756,rs137853007,rs137853011,rs141568342,rs142763740,rs143767430,rs1565909,rs17352824,rs17879961,rs1800057,rs1801516,rs180177083,rs180177099,rs180177100,rs180177110,rs180177132,rs180177133,rs180177142,rs180177143,rs1964010,rs1980271,rs200432447,rs200928781,rs201523522,rs201963507,rs202206540,rs2132096,rs2132097,rs2172809,rs2177034,rs2588809,rs273899695,rs273901751,rs28488925,rs28603076,rs28653564,rs28783127,rs28785823,rs28869016,rs28869596,rs28875243,rs28895925,rs28897672,rs28897696,rs28897743,rs28897756,rs28897758,rs28904921,rs28909982,rs34136446,rs34803094,rs34961555,rs35000866,rs35452111,rs35608460,rs35850753,rs36014997,rs371638537,rs376603775,rs397507239,rs397507320,rs397507331,rs397507333,rs397507404,rs397507582,rs397507686,rs397507713,rs397507762,rs397507868,rs397507943,rs397508034,rs397509267,rs397515635,rs398122546,rs398122602,rs398122749,rs398122772,rs41293455,rs41293459,rs41293461,rs41293463,rs41293465,rs41293477,rs41293497,rs41293511,rs41293513,rs431825343,rs45444999,rs45476495,rs45553935,rs45580035,rs4693090,rs515726117,rs526064,rs532480170,rs536907995,rs538078,rs543318,rs555348,rs555607708,rs55770810,rs562723,rs564652222,rs568978,rs587776408,rs587776410,rs587776411,rs587776416,rs587776527,rs587779815,rs587779818,rs587779836,rs587779844,rs587779846,rs587779865,rs587779869,rs587779872,rs587780174,rs587780210,rs587781269,rs587781347,rs587781598,rs587781658,rs587781730,rs587781776,rs587781905,rs587782005,rs587782070,rs587782081,rs587782173,rs587782198,rs587782240,rs587782245,rs587782292,rs587782471,rs587782652,rs587782680,rs587782719,rs59644543,rs5997390,rs62625303,rs62625308,rs6535478,rs6834006,rs6838225,rs6844460,rs72552322,rs72755295,rs730881304,rs730881348,rs730881359,rs730881391,rs730881394,rs730881699,rs730881700,rs730881701,rs730882166,rs73161324,rs747070579,rs747727055,rs748634900,rs749036865,rs75096777,rs758588019,rs760502479,rs761494650,rs762083530,rs763470424,rs764925691,rs7663427,rs7666601,rs7671387,rs7691492,rs7694435,rs769523686,rs770641163,rs772821016,rs774175654,rs776294034,rs778269655,rs779278389,rs780905851,rs78378222,rs786201675,rs786201689,rs786201693,rs786201957,rs786202239,rs786202497,rs786203008,rs786203053,rs786203054,rs786203272,rs786203370,rs786203488,rs786203606,rs786203714,rs786204273,rs786204433,rs786204751,rs796389290,rs80356885,rs80356898,rs80356929,rs80356978,rs80356991,rs80357018,rs80357243,rs80357426,rs80357508,rs80357516,rs80357522,rs80357542,rs80357572,rs80357573,rs80357687,rs80357723,rs80357729,rs80357760,rs80357813,rs80357853,rs80357868,rs80357888,rs80357906,rs80357939,rs80357960,rs80357968,rs80357970,rs80357971,rs80358094,rs80358504,rs80358523,rs80358533,rs80358556,rs80358579,rs80358620,rs80358710,rs80358721,rs80358810,rs80358824,rs80358843,rs80358972,rs80358981,rs80359003,rs80359022,rs80359031,rs80359064,rs80359075,rs80359076,rs80359099,rs80359140,rs80359144,rs80359159,rs80359200,rs80359212,rs80359230,rs80359272,rs80359277,rs80359281,rs80359282,rs80359283,rs80359308,rs80359312,rs80359315,rs80359316,rs80359328,rs80359340,rs80359351,rs80359356,rs80359371,rs80359383,rs80359388,rs80359391,rs80359395,rs80359401,rs80359405,rs80359438,rs80359444,rs80359460,rs80359461,rs80359462,rs80359470,rs80359479,rs80359480,rs80359484,rs80359487,rs80359496,rs80359503,rs80359505,rs80359507,rs80359511,rs80359512,rs80359520,rs80359550,rs80359596,rs80359598,rs80359601,rs80359603,rs80359604,rs80359623,rs80359629,rs80359636,rs80359646,rs80359659,rs80359679,rs80359705,rs80359718,rs80359730,rs80359743,rs80359753,rs80359763,rs80359775,rs8042593,rs813298,rs863225466,rs864622389,rs864622649,rs876659258,rs879255306,rs9284657,rs9683689,rs971539,rs9997925 | 0.000008 |
| Resolution of D-loop Structures through Holliday Junction Intermediates | DNA Repair | *ATM,BRCA1,BRCA2,EXO1,PALB2,RAD51B* | rs11571658,rs11571833,rs11624333,rs118203998,rs118203999,rs121434219,rs1800057,rs1801516,rs180177083,rs180177099,rs180177100,rs180177110,rs180177132,rs180177133,rs180177142,rs180177143,rs201523522,rs201963507,rs202206540,rs2588809,rs273899695,rs273901751,rs28897672,rs28897696,rs28897743,rs28897756,rs28897758,rs28904921,rs371638537,rs376603775,rs397507239,rs397507320,rs397507331,rs397507333,rs397507404,rs397507582,rs397507686,rs397507713,rs397507762,rs397507868,rs397507943,rs397508034,rs397509267,rs397515635,rs398122546,rs398122602,rs398122749,rs398122772,rs41293455,rs41293459,rs41293461,rs41293463,rs41293465,rs41293477,rs41293497,rs41293511,rs41293513,rs431825343,rs45444999,rs45476495,rs45553935,rs45580035,rs515726117,rs532480170,rs55770810,rs564652222,rs587776408,rs587776410,rs587776411,rs587776416,rs587776527,rs587779815,rs587779818,rs587779836,rs587779844,rs587779846,rs587779865,rs587779869,rs587779872,rs587780210,rs587781347,rs587781598,rs587781658,rs587781730,rs587781776,rs587781905,rs587782005,rs587782081,rs587782173,rs587782198,rs587782240,rs587782292,rs587782652,rs587782680,rs587782719,rs62625303,rs62625308,rs72755295,rs730881304,rs730881348,rs730881359,rs730881391,rs730881394,rs730882166,rs747070579,rs747727055,rs748634900,rs749036865,rs75096777,rs758588019,rs762083530,rs763470424,rs764925691,rs769523686,rs770641163,rs772821016,rs776294034,rs778269655,rs779278389,rs780905851,rs786201675,rs786201689,rs786201693,rs786201957,rs786202239,rs786203008,rs786203054,rs786203272,rs786203370,rs786203488,rs786203606,rs786203714,rs786204273,rs786204433,rs786204751,rs80356885,rs80356898,rs80356929,rs80356978,rs80356991,rs80357018,rs80357243,rs80357426,rs80357508,rs80357516,rs80357522,rs80357542,rs80357572,rs80357573,rs80357687,rs80357723,rs80357729,rs80357760,rs80357813,rs80357853,rs80357868,rs80357888,rs80357906,rs80357939,rs80357960,rs80357968,rs80357970,rs80357971,rs80358094,rs80358504,rs80358523,rs80358533,rs80358556,rs80358579,rs80358620,rs80358710,rs80358721,rs80358810,rs80358824,rs80358843,rs80358972,rs80358981,rs80359003,rs80359022,rs80359031,rs80359064,rs80359075,rs80359076,rs80359099,rs80359140,rs80359144,rs80359159,rs80359200,rs80359212,rs80359230,rs80359272,rs80359277,rs80359281,rs80359282,rs80359283,rs80359308,rs80359312,rs80359315,rs80359316,rs80359328,rs80359340,rs80359351,rs80359356,rs80359371,rs80359383,rs80359388,rs80359391,rs80359395,rs80359401,rs80359405,rs80359438,rs80359444,rs80359460,rs80359461,rs80359462,rs80359470,rs80359479,rs80359480,rs80359484,rs80359487,rs80359496,rs80359503,rs80359505,rs80359507,rs80359511,rs80359512,rs80359520,rs80359550,rs80359596,rs80359598,rs80359601,rs80359603,rs80359604,rs80359623,rs80359629,rs80359636,rs80359646,rs80359659,rs80359679,rs80359705,rs80359718,rs80359730,rs80359743,rs80359753,rs80359763,rs80359775,rs863225466,rs864622389,rs864622649,rs876659258,rs879255306 | 0.000009 |

**References**

1 Mavaddat, N. *et al.* Polygenic Risk Scores for Prediction of Breast Cancer and Breast Cancer Subtypes. *The American Journal of Human Genetics* **104**, 21-34 (2019). https://doi.org:10.1016/j.ajhg.2018.11.002

2 Michailidou, K. *et al.* Association analysis identifies 65 new breast cancer risk loci. *Nature* **551**, 92-94 (2017). https://doi.org:10.1038/nature24284

3 Pereira, B. *et al.* The somatic mutation profiles of 2,433 breast cancers refines their genomic and transcriptomic landscapes. *Nat Commun* **7**, 11479 (2016). https://doi.org:10.1038/ncomms11479
